# Large-Scale Screening of a Targeted *Enterococcus faecalis* Mutant Library Identifies Envelope Fitness Factors

**DOI:** 10.1371/journal.pone.0029023

**Published:** 2011-12-15

**Authors:** Lionel Rigottier-Gois, Adriana Alberti, Armel Houel, Jean-François Taly, Philippe Palcy, Janet Manson, Daniela Pinto, Renata C. Matos, Laura Carrilero, Natalia Montero, Muhammad Tariq, Harma Karsens, Christian Repp, Andrea Kropec, Aurélie Budin-Verneuil, Abdellah Benachour, Nicolas Sauvageot, Alain Bizzini, Michael S. Gilmore, Philippe Bessières, Jan Kok, Johannes Huebner, Fatima Lopes, Bruno Gonzalez-Zorn, Axel Hartke, Pascale Serror

**Affiliations:** 1 INRA, UMR1319 Micalis, Jouy-en-Josas, France; 2 AgroParisTech, UMR Micalis, Jouy-en-Josas, France; 3 INRA-UR1077-MIG, Jouy-en-Josas, France; 4 Departments of Ophthalmology and Microbiology and Molecular Genetics, Harvard Medical School, Boston, Massachusetts, United States of America; 5 Instituto de Biologia Experimental e Tecnológica, Oeiras, Portugal; 6 Departamento de Sanidad Animal, Facultad de Veterinaria and VISAVET, Universidad Complutense de Madrid, Madrid, Spain; 7 Molecular Genetics, University of Groningen, Centre for Life Sciences, Groningen, The Netherlands; 8 Division of Infectious Diseases, University Hospital Freiburg, Freiburg, Germany; 9 Lab of Environmental Microbiology, IBFA, Université de Caen, Caen, France; 10 Instituto de Tecnologia Química e Biológica, Universidade Nova de Lisboa, Oeiras, Portugal; University of Liverpool, United Kingdom

## Abstract

Spread of antibiotic resistance among bacteria responsible for nosocomial and community-acquired infections urges for novel therapeutic or prophylactic targets and for innovative pathogen-specific antibacterial compounds. Major challenges are posed by opportunistic pathogens belonging to the low GC% Gram-positive bacteria. Among those, *Enterococcus faecalis* is a leading cause of hospital-acquired infections associated with life-threatening issues and increased hospital costs. To better understand the molecular properties of enterococci that may be required for virulence, and that may explain the emergence of these bacteria in nosocomial infections, we performed the first large-scale functional analysis of *E. faecalis* V583, the first vancomycin-resistant isolate from a human bloodstream infection. *E. faecalis* V583 is within the high-risk clonal complex 2 group, which comprises mostly isolates derived from hospital infections worldwide. We conducted broad-range screenings of candidate genes likely involved in host adaptation (e.g., colonization and/or virulence). For this purpose, a library was constructed of targeted insertion mutations in 177 genes encoding putative surface or stress-response factors. Individual mutants were subsequently tested for their i) resistance to oxidative stress, ii) antibiotic resistance, iii) resistance to opsonophagocytosis, iv) adherence to the human colon carcinoma Caco-2 epithelial cells and v) virulence in a surrogate insect model. Our results identified a number of factors that are involved in the interaction between enterococci and their host environments. Their predicted functions highlight the importance of cell envelope glycopolymers in *E. faecalis* host adaptation. This study provides a valuable genetic database for understanding the steps leading *E. faecalis* to opportunistic virulence.

## Introduction

Enterococci are ubiquitous low-GC percent Gram-positive bacteria. The two clinically predominant species *E. faecalis* and *E. faecium* are natural members of the digestive microbiota in humans. Interestingly, they are also found as members of the natural microflora of a variety of fermented food products [1], [2]. While not regarded as particularly virulent, both species have emerged as major causes of nosocomial infections. They are the second most common cause of nosocomial bloodstream infections in the United States [3] and the fourth in Europe (http://www.earss.rivm.nl/). Enterococci cause disease mainly in patients i) undergoing prolonged antibiotic treatments, ii) with severe underlying diseases, and iii) with an impaired immune system (e.g., after solid organ transplantation and/or immunosuppressive therapy) [4]. They are mainly responsible for urinary tract infections, bacteremia, wound infections, and endocarditis [5], with *E. faecalis* accounting for 60 to 80% of all enterococcal infections [5], [6].

Enterococci are intrinsically resistant to a broad range of the antibiotics commonly used in the hospital setting, which in part explains their high prevalence in nosocomial infections [7]. Their acquired resistance to most antibiotics used in the hospital, including acquired vancomycin resistance, is most worrisome since it limits therapeutic alternatives against multiply resistant strains. Furthermore, enterococci are considered a reservoir for antibiotic resistance genes, as may be exemplified by their ability to transfer vancomycin resistance to methicillin resistant *Staphylococcus aureus*, for which vancomycin remains the last therapeutic alternative [8], and to other bacterial genera [9].

As part of the normal intestinal microbiota, *E. faecalis* can cross the intestinal epithelial barrier and enter the bloodstream [10], [11], [12], [13]. Macrophages have been suggested to serve as vehicles for enterococcal invasion and dissemination [10]. To survive within macrophages, bacteria must adapt to this intracellular environment. They have to cope with the host cell stress arsenal of antimicrobial defenses, including the production of reactive oxygen species and the low pH of the phagosome [14]. The enterococcal adaptation and virulence factors remain only partially understood.

Over the last fifteen years, a number of studies have attempted to identify *E. faecalis* virulence factors (for review [15], [16], [17]). The putative *E. faecalis* virulence genes include the surface adhesins Esp, AS and Ace, the secreted toxin cytolysin Cyl, the secreted proteases GelE and SrpE, two cell wall polysaccharides, Cps and Epa, and the internalin-like protein ElrA. Transcriptional regulators found to be involved in *E. faecalis* virulence are the Agr-like Fsr system [18], EtaRS [19] and HypR [20], [21]. In addition, analyses of the *E. faecalis* V583 genome and pathogenicity island of *E. faecalis* strain MMH594, two strains of the high-risk enterococcal clonal complex 2 (CC2) responsible for infections in hospitalized patients, led to the identification of a number of genes that may also encode putative *E. faecalis* virulence factors [22], [23]. These entail putative adhesins and invasins, exoenzymes, proteases, and surface and extracellular proteins. However, these predictions need to be validated by functional studies, which may have been limited by difficulties in genetic manipulation of the encapsulated V583 strain, which is resistant to multiple antibiotics and carries three replicating plasmids [24], [25].

The aim of our study was to identify new *E. faecalis* host adaptation factors allowing this bacterium to be an opportunistic pathogen in humans. Therefore, we used the published V583 genome sequence to compile a list of genes with potential involvement in virulence. We then designed a mutational system that was used in a systematic post-genomic approach for inactivation of 177 of the selected genes of *E. faecalis*. The mutants were subjected to a series of assays that examined their ability to resist several life-style-related conditions. These combined efforts led to the identification of numerous genes playing a role under these conditions.

## Results

### Target gene selection, selection of a plasmid-free derivative of *E. faecalis* V583 and production of a library of targeted mutants

Analysis of the *E. faecalis* V583 genome led to the selection of 223 putative virulence gene candidates encoding i) surface exposed proteins such as cell wall-anchored proteins, lipoproteins and transporters and proteins involved in surface polysaccharides or lipopolysaccharides synthesis; ii) putative transcriptional regulators; iii) proteins of unknown function identified as specific to *E. faecalis*; iv) enzymes involved in carbohydrate metabolism; v) proteins involved in stress resistance; or vi) proteins similar to known virulence factors, such as hyaluronidase, internalin-like protein, and proteases. A percentage of 85 and 15 of the selected genes are part of the core genome or located on mobile genetic elements, respectively, including 17 genes that are present in the pathogenicity island [23].

To avoid problems related to the presence of replicating plasmids we selected a plasmid-cured derivative of strain V583, as described in the Materials and Methods section, named VE14089. This strain has not undergone major DNA rearrangements, as indicated by DNA microarray analysis, pulsed field gel electrophoresis, Southern blotting, long-range PCR and DNA sequencing analyses, with one exception: a 20.5-kb region of pTEF1 (from *efa0063* up to *efa0006*) is integrated between chromosomal genes *ef3209* and *ef3210*, and is flanked by two copies of IS*1216*. As expected, the cured strain was sensitive to erythromycin and gentamicin and related macrolides and aminoglycosides resulting from plasmid loss, but remains as resistant as the wild-type strain V583 to other antibiotics tested. We compared both strains for several phenotypes. The cured strain demonstrated a growth defect in comparison to the parental strain which was more pronounced under aerobic than anaerobic conditions. However, the cured strain had an only minor reduced ability to cause lethality in the *Galleria mellonella* virulence model (Fig. S1), indicating that strain VE14089 remained relevant for *E. faecalis* genetic studies on virulence.

The system for generating targeted chromosomal insertions by homologous recombination is based on the conditional replication of two plasmids [26]. Plasmid pVE14218 (RepA−) is a cloning vector with a tetracycline marker used to clone internal fragments of each gene to be inactivated. Plasmid pG+host3 is a thermosensitive helper plasmid that provides the RepA replication protein to pVE14218 at permissive temperature. The pVE14218 derivative plasmids were integrated in the targeted loci as described in Materials and Methods. Thus, from a total of 223 gene disruptions attempted on candidates, 177 isogenic V583-derived mutants were successfully obtained, which targeted genes in 14 of the 20 gene role categories as defined by the JCVI CMR database (Table S1). Unsuccessful mutagenesis could be explained either by an essential role of the targeted genes in the conditions used and/or their poor recombinogenic potential. One-third of the mutants target functions involved in cell envelope and in transport. The whole mutant library was then submitted to several test conditions. The target genes were assigned as affecting four categories of phenotypes related to host adaptation, namely: (i) stress and antibiotic resistance, (ii) resistance to opsonophagocytosis, (iii) adherence to Caco-2 epithelial cells and (iv) virulence in a *Galleria mellonella* insect model.

### Mutant growth behavior, fitness in stressing environment and resistance to antibiotics

Growth behaviour and fitness under oxidative stress conditions have been tested for the entire *E. faecalis* mutant library using microtiter plate assays. The analyses evidenced more or less important growth defects under non-stress condition in 10 of the mutants (Table S1). The strongest effects were observed in strains with mutations in genes encoding putative glycosyltransferases (EF2167, EF2170 and EF2196). The majority of the strains (125) showed no significant differences in sensitivity to exposure to H_2_O_2_ compared to the parental strain. The 52 mutants that were more sensitive to H_2_O_2_ were grouped in three categories with respect to the degree of their sensitivities (slightly, highly and extremely sensitive) to the stressing agent (Table S2). The most sensitive mutants were affected in genes encoding transcriptional regulators (EF0073 and EF1599) belonging to the Cro/CI family, EF0107 present in the Arginine deiminase operon [27] and EF1525, one of the three Fur-paralogs, proteins implicated in envelope metabolism (EF1172, EF1746, EF2170, EF2180, EF2196, EF2198), a paralog of the virulence factor Gls24 (EF0604 encoded by the PAI), an ABC transporter (EF1408) and the antioxidant activity and virulence factor MsrB (EF3164) [28]. Of note, no mutant more resistant to H_2_O_2_ than the parental strain was identified.

Antibiotic resistance of the VE14089 strain and all its isogenic mutants was determined for 34 antibiotics that, together, cover all known mechanisms of antibiotic action (Table S3). More than 88% of the mutants showed no modification of their antibiotic resistance, including 7 mutants in genes annotated as having a role in drug resistance (*ef0420*, *ef0785*, *ef1042*, *ef1814*, *ef1943*, *ef2068* and *ef2772*; see Table S3). Of the 21 mutants displaying a modified antibiotic resistance profile, increased sensitivity to ceftriaxone was observed in a mutant with an interrupted *npr*-gene (*ef1211*). This gene encodes the well characterized NADH peroxidase of *E. faecalis* and its main cellular role is to protect cells against internal metabolically-derived oxidative stress [29]. Two mutants putatively affected in the cell envelope were more sensitive to fusidic acid (*ef1746* and *ef2181*); this might relate to changes in cell permeability. Five mutants showed increased resistance to amoxicillin and 2 to chloramphenicol; the targeted genes are predicted to have roles in signal transduction (n = 3), in cell envelope (n = 2), in cellular processes (n = 1) and in protein fate (n = 1). Among the low number of mutants that showed altered antibiotic sensitivity, we noted a significant enrichment of genes affected in the categories “cell envelope” and “signal transduction” compared to the representation of these two categories in the entire mutant collection (Table 1).

**Table 1:** Percentage of mutants in each JCVI role category.

JCVI role category	% mutant collectionn = 177	% mutant affected in the following phenotype			
		Sensitivity to H_2_O_2_	Sensitivity to antibiotics	Opsonophago-cytosis assay	Virulence in *G. mellonella*
		n = 52	n = 21	n = 14	n = 57
Biosynthesis of cofactors, prosthetic groups, and carriers	0.6	1.9	-	-	-
Cell envelope	24.3	25.0	38.1*	71.4*	26.3
Cellular processes	7.9	7.7	9.5	-	3.5
Energy metabolism	6.8	5.8	14.3	-	5.3
Fatty acid and phospholipid metabolism	4.5	-	-	-	1.8
Hypothetical proteins	9	9.6	-	7.1	7
No Data	5.6	9.6	4.8	-	7
Protein fate	4.5	1.9	4.8	-	5.3
Protein synthesis	1.1	1.9	-	-	-
Regulatory functions	14.1	15.4	4.8	14.3	21.1^a^
Signal transduction	2.8	-	14.3*	-	3.5
Transcription	0.6	-	-	-	-
Transport and binding proteins	15.3	17.3	9.5	7.1	19.3
Unknown function	2.8	3.8	-	-	-

a Significant enrichment compared to the frequency in the mutant collection.

### Resistance to opsonophagocytosis

Opsonic killing of bacteria constitutes a reliable *in vitro* surrogate of protective immunity *in vivo* and occurs in the presence of specific antibodies, complement, and phagocytes [30]. Different structures on the bacterial surface can be the target of opsonic antibodies and the loss of such structures will render the mutant more resistant to opsonic killing. Specific mechanisms of the bacteria to withstand the protective immune response of the host can be observed in the same assay by identifying mutants that become hyper-susceptible to opsonic killing when one of these factors is eliminated [31].

Sera raised against *E. faecalis* V583 were tested for their opsonic killing activity against the various mutant strains. From 177 mutants tested, only 3 were more resistant to opsonophagocytosis while 11 were more sensitive (1 with complement alone and 10 without antiserum, see Table S4). Among these, 10 (9 sensitive and 1 resistant mutant) carried mutations in genes predicted to have a role in the cell envelope. In contrast to the VE14089 strain, most of these mutants were efficiently killed by a combination of polymorphonuclear neutrophils (PMNs) with complement, while the additional effect of antibody opsonization was negligible. Two mutants targeted in genes probably involved in wall-teichoic acid synthesis (*ef1172*, *ef1173*) were susceptible to complement and PMNs. Three mutants displayed significantly higher resistance to opsonic killing compared to the wild-type. Two were mutated in adjacent transcriptional regulator genes *ef0600*–*ef0601*, which are part of the pathogenicity island of strain V583 enriched in CC2 complex [32] and both encode regulators of the TetR family of transcriptional regulators. One strain mutated in gene *ef2490* (*cpsF*) was also more resistant to opsonic killing. CpsF is responsible for the sero-specificity of serotype C to which VE14089 as a derivative of V583 belongs [33]. It was shown previously that removal of CpsF changes this strain to sero-group D thereby abrogating the activity of anti-serotype C antibodies [34]. Disruption of capsule genes *ef2491* (*cpsE*) and *ef2492* (*cpsD*) did not increase susceptibility to killing by complement and PMNs. This result may be explained by the fact that LTA unmasked by capsular polysaccharide also protects *E. faecalis* from opsonic killing [33], [34], [35].

### Adhesion to Caco-2 epithelial cells

Non-invasive bacteria that colonize the gastrointestinal tract, such as entero-hemorrhagic *Escherichia coli* (EHEC), adhere tightly to epithelial cells and promote pedestal formation [36]. Although there is no consensus about the capacity of enterococci to invade epithelial cells, adherence to such cells is likely to contribute to enterococcal pathogenesis. To evaluate this ability in the 177 *E. faecalis* mutants, we used a 96-well microtiterplate screening model for adherence of *E. faecalis* to cells of the human colon carcinoma Caco-2 epithelial cell-line. This cell-line has already proven to be a valuable model for testing adherence of *E. faecalis* to epithelial cells [37], [38].

Eighty-four percent of the mutants were not modified with respect to their adhesion to the intestinal epithelial cells. Four mutants displayed a decreased ability to adhere (Table S5); the interrupted genes have predicted roles in signal transduction (PTS system; *ef0553*), in cell envelope (*ef0252*) and in cellular processes (*ef1076*). In one of the adhesion-defective mutants, a hypothetical protein (EF0876) was affected that carries a domain found in the streptococcal regulator Mga, which is known to regulate production of adhesins in group A streptococci [39]. Interestingly, 24 mutants showed increased adhesion to the epithelial cells, 9 of which carried mutations in genes with a predicted role in the cell envelope, such as polysaccharide biosynthesis or degradation.

### Virulence in the *Galleria mellonella* insect model

Increasing interest in using *G. mellonella* as a surrogate model to study virulence of various microorganisms led us to compare the virulence of the 177 mutant strains with that of the parental strain. One third of the mutants (n = 57) were modified in virulence in this model: 49 showed decreased virulence and 8 were more virulent (Fig. S2, Table S6). Most of the mutations of both mutant categories are in genes potentially involved in cell envelope metabolism, regulatory functions and transport. Eight genes encode proteins of unknown function, including 3 of which the products are predicted to locate at the cell envelope (*ef1420*, *ef2250* and *ef2276*). A slight enrichment in the category “Regulatory Function” is observed (Table 1). The diversity of the regulator classes affecting *E. faecalis* virulence indicates that this process relies on a large number of functions in this model. Bacterial virulence in insects such as the Lepidoptera *G. mellonella* lies in the ability to resist the innate immune response, which includes the action of lysozyme, antimicrobial peptides and reactive oxygen and nitrogen species such as hydrogen peroxide and nitric oxide [40]. Among the attenuated mutants, some carry mutations in genes well known to participate in resistance to antimicrobial peptides of *E. faecalis* or other bacteria. These include genes involved in the dealanylation of LTA (*ef2746* and *ef2748*), in the biosynthesis of teichoic acids (*ef1172* and *ef1175*), in membrane phospholipid synthesis (*ef1027*, an *mprF*-like gene) or potential efflux pumps (*ef0785*, *ef1760*, *ef1814* and *ef2068*).

### Combined phenotypic analysis of the *E. faecalis* mutant library

**Figure 1:**
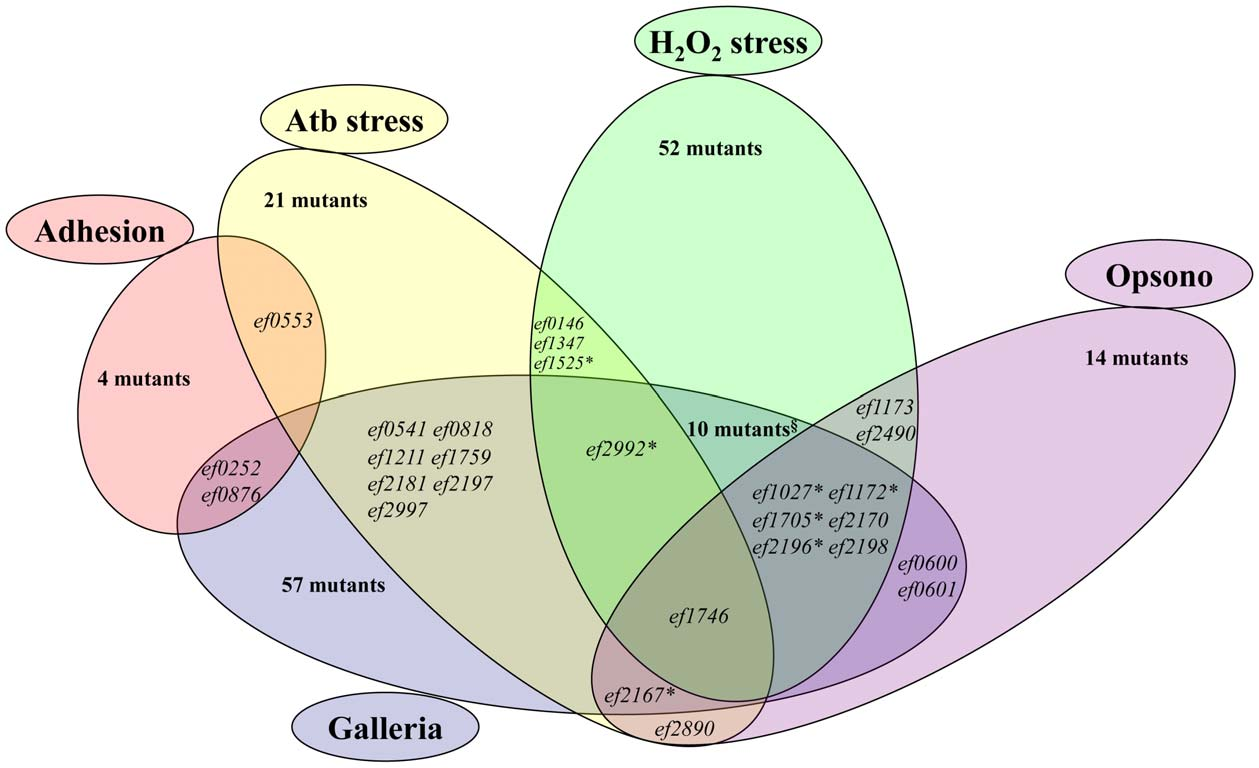
Venn diagram representation of the results of the five phenotypic tests performed on the mutants obtained in this study. Only genes that are common to at least two phenotypes are listed. The increased adhesion phenotype was not included due to difficulties in constructing the Venn diagram with these data. Stars indicate mutants with increased adhesion phenotype. **^§^** The 10 mutants combining phenotypes for H_2_O_2_ stress and virulence are not shown due to lack of space in the figure. They were inactivated in *ef0073*, *ef0465*, *ef0814*, *ef1212*, *ef1420*, *ef1493*, *ef1741*, *ef1760*, *ef1798* or *ef2442*.

All the data collected for each of the mutants constructed in this study were entered in an easy-access phenotypic database (http://genome.jouy.inra.fr/phenotype/). From the 177 mutant strains, more than 1/3 showed no detectable phenotype and 36% (n = 64), 19% (n = 34) and 6% (n = 11) were tested positive for one, two and more than two phenotypes, respectively. A Venn diagram was constructed according to the intersection of predicted genes in the 5 phenotyping tests, with the exception of the increased adhesion phenotype (Fig. 1). In this analysis, a handful of phenotype combinations were observed, the most frequent being the combination of resistant to H_2_O_2_ and virulent in *G. mellonella*. Noticeably, the majority (13/14) of the mutants affected in the opsonophagocytosis assay displayed at least another phenotype, with sensitivity to H_2_O_2_ and virulent in *G. mellonella* being the most prevalent. Furthermore, half of the mutants with modified antibiotic resistance are affected in virulence. One mutant exhibited four phenotypes (*ef1746*) while 8 mutants displayed three phenotypes (*ef1027*, *ef1172*, *ef1705*, *ef2170*, *ef2196*, *ef2198*, *ef2167*, *ef2992*). The affected genes mostly encode proteins with predicted roles in cell envelope metabolism, in polysaccharide synthesis or degradation, or in transport or substrate binding.

## Discussion

Bacterial pathogens that invade the animal host are faced with various hostile conditions aimed at their elimination, *via* products of the host innate and adaptive immune responses, and in some cases, antibiotics. Success of the pathogen requires its evolving capacity to withstand host defenses. Various global approaches have been applied to search for bacterial genes that increase pathogenicity, which include screening of random mutant libraries in animal models and *in vitro* tests mimicking a specific stage of the infection (e.g. challenges with H_2_O_2_, acid or antimicrobial peptides, growth in a biofilm), and *in vivo* mRNA transcriptome analyses [41], [42]. So far, large-scale screening for host adaptation factors has rarely been applied to *E. faecalis*, and strain V583, which belongs to clonal complex 2, has been thus far overlooked [43], [44], [45], [46]. To deepen our knowledge of factors involved in enterococcal virulence, we constructed a bank of 177 targeted mutants in V583 genes that were chosen on the basis of their potential roles in host fitness. This first large set of isogenic *E. faecalis* V583 mutants constitutes a unique resource for identifying genes, and functions that are important under various *E. faecalis* life-style related conditions (e. g. biofilm formation, exposure to biocides, and interaction with host cells). This work should thus help elucidate the mechanisms involved in *E. faecalis* infection.

**Figure 2:**
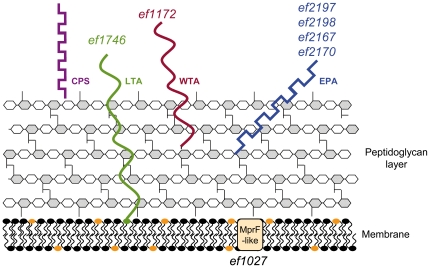
Schematic representation of *E. faecalis* V583 cell envelope, showing functions involved in pleiotropic stress protection. The *E. faecalis* V583 cell envelope includes the peptidoglycan-anchored teichoic acid (WTA; burgundy), the membrane-anchored lipoteichoic acid (LTA; green), the rhamnopolysaccharide (EPA; blue), and a capsular polysaccharide (CPS; purple). Names of genes predicted to participate in glycopolymer synthesis are indicated with the cognate colour code. Membrane protein MprF-like is depicted in light orange.

Our approach consisted of individual screening of all mutants for phenotypes related to the enterococcal *in vivo* lifestyle: antibiotic resistance, oxidative stress resistance, adherence, and infection. Almost two thirds of the mutants (n = 109) exhibited a phenotype under the conditions tested, thus supporting the relevance of our initial choice of target genes. Our next step is to confirm the association between phenotype and the target mutations, so as us to rule out possible polar effects or secondary mutations among the mutated strains. Importantly, the majority of these mutants (n = 91 out of 109) were affected in oxidative stress responses and/or in virulence. A minority (n = 32 out of 109) displayed modified resistance to opsonophagocytosis or to antibiotics, but most of these were multiply affected. A total of 45 mutants displayed multiple phenotypes. The observed phenotypes correlate with the importance for the successful bacterial pathogen to overcome phagocytosis and the cognate oxidative burst, as well as cell wall stress caused by the action of lysozymes and antimicrobial peptides [47], [48]. The significant overlap between antibiotic resistance and virulence phenotypes suggests that factors for intrinsic antibiotic resistance are probably involved in general stress response and therefore contribute to a better fitness in the host. Pleiotropic phenotypes associated with mutations in surface functions underline the role of the cell envelope in combating external stress conditions encountered in the host. As discussed below, glycopolymers, rather than proteins, emerged from this study as the main structural group responsible for *E. faecalis* defense against multiple external assaults (Fig. 2).

Our and previous work point to a major role of cell wall glycopolymers including polysaccharides in protection against the host responses and in bacterial colonization of mucosal surfaces in both commensals and pathogens [49]. The most abundant enterococcal glycopolymers are lipid-attached lipoteichoic acids (LTA), peptidoglycan-attached wall teichoic acids (WTA) [50] and glycolipids [31], [38]. The best studied enterococcal cell wall polysaccharides are a rhamnopolysaccharide (Epa; this polysaccharide is the presumed product of the *epa* locus) and a type-specific capsular polysaccharide (Cps) [51], [52], [53], [54]. Results of our mutants affecting different glycopolymers are presented according to the class of glycopolymers that are affected.

GalU (EF1746; uridine diphosphoglucose pyrophosphorylase) converts glucose-1 phosphate to UDP-glucose, an essential substrate for LTA glycolipid anchor synthesis and a precursor for the synthesis of sugar polymers such as exocellular (lipo)polysaccharides [55]. Inactivation of *galU* gave a pleiotropic effect, with 4 detected phenotypes. GalU was previously implicated in *Streptococcus pneumoniae* capsular polysaccharide biosynthesis and virulence [56]. The present results on the role of *galU* in *E. faecalis* suggest that this enzyme may be of general importance among the opportunist pathogens for host adaptation.

WTA and LTA are implicated in attachment to epithelial and endothelial surfaces and to biomaterials, and in host cell invasion, as reported in numerous Gram-positive bacteria [57]. In our work, inactivation of TagB (EF1172), a glycerophosphotransferase required for WTA biosynthesis, resulted in a strain with multiple phenotypes. In view of this predicted function of *tagB*, the absence of WTA in the *tagB* mutant should lead to an increased negative bacterial net charge. A consequence of surface charge modifications is an altered susceptibility to cationic antimicrobial peptides [50], [58]. Such consequences are in keeping with the observed *tagB* multiple phenotypes in *E. faecalis*. Interestingly, inactivation of *ef1027*, encoding a putative MprF-like protein also led to multiple phenotypes. MprF is a membrane protein catalyzing the modification of the negatively charged lipid phosphatidylglycerol with L-lysine or L-alanine, thus neutralizing the membrane surface [59]. These modifications could explain previous observations of attenuated reduced virulence in the *mprF* mutant in *S. aureus*, *Listeria monocytogenes* and *Mycobacterium tuberculosis* [60], [61], [62]. As with the *tagB* mutant, loss of *mprF* might result in an increased negative membrane charge, and might consequently contribute to cationic antimicrobial peptides sensitivity and more generally to host fitness.

The *epa* operon required for rhamnopolysaccharide biosynthesis involves V583 genes *ef2198* to *ef2177*, as extrapolated from assignments in *E. faecalis* strain OG1RF (in which *epa* comprises 18 genes, *epaA* to *epaR* [51], [54], [63]. *E. faecalis* V583 mutants that mapped in the *epa* region (*ef2196* and *ef2198*) or adjacently (*ef2167* and *ef2170*) all displayed multiple phenotypes In OG1RF, mutants in *epaB* (*ef2197*) and *epaE* (*ef2194*) were attenuated for virulence in a mouse peritonitis model [64], more susceptible to PMN-killing [53], and were deficient in translocation across enterocyte-like T84 cells [65] and biofilm formation [54], [66]. The pleiotropic effects of *ef2196* and *ef2198*, all tested in parallel, give support for a key role of *epa* in host adaptation. The fact that the strongest *in vitro* growth defect was observed when mutations occurred in this locus supports the idea that the *epa* genes have a general role in the physiology of the bacteria and represent fitness rather than *bona fide* virulence factors. The absence of potential transcriptional terminators separating genes *ef2167* and *ef2170* from *epa*, coupled with the identical phenotypes obtained for the four mutants, leads us to suggest that the *epa* locus in V583 spans genes *ef2198* to *ef2164*. Genes *ef2167* and *ef2170* are enriched in isolates of the high-risk enterococcal clonal complex 2 [32], [67], [68], and we predict their products are involved in structural diversity of Epa.

Our studies point to the general importance of glycopolymers for defense against commonly encountered stress conditions. Based on the successful development of vaccine strategies using glycopolymers of several pathogens [58], and on promising results with enterococcal LTA [69], [70], it is tempting to propose *E. faecalis* glycopolymers as vaccination targets.

Mutants inactivated for inorganic ion transport systems or their corresponding regulatory functions also displayed pleiotropic phenotypes. As described in other bacterial species, phosphate uptake systems may additionally transport metals [71], [72], [73]. Since metals can increase oxidative stress [74], sensitivity of two mutants in *ef1705* and *ef2442* to oxidative stress could result from disrupted metal homeostasis. Maintenance of metal homeostasis appears to be important for resistance to harsh conditions found in the host. Fur-like regulators control intracellular concentrations of iron, zinc or other metals in many bacteria, and may impact on host fitness [75]. *E. faecalis* strains inactivated for these regulators (EF1525, EF1585 and EF2417) gave results similar to those with the ion transport mutants, suggesting that these mutations may affect common functions. Of the 3 Fur family transcriptional regulators tested, only PerR (EF1585) has been studied in *E. faecalis* JH2-2 [76]. Inactivation in strain V583 of EF1585 (PerR) or EF2417 attenuated virulence in the *G. mellonella* insect model, and deletion of EF1525 increased both sensitivity to H_2_O_2_ and resistance to gentamycin. These results suggest that *E. faecalis* paralogs of Fur-like proteins somehow have a dedicated role in host fitness. As the paralogs could compensate each other, at least partially from overlapping phenotypes, they could contribute to the flexibility of the cell to adapt to different environmental conditions.

In conclusion, this study provides the first large collection of isogenic mutants of CC2 isolate V583 available to the scientific community, and the first large-scale functional characterization regarding multiple phenotypes related to *E. faecalis* host adaptation. Our study has highlighted the major and pleiotropic role of *E. faecalis* cell wall glycopolymers under conditions that mimic host adaptation, such as adhesion, resistance to immune response, colonisation, and signalling. Further work will allow us to characterize selected cell envelope-related fitness factors and to identify how they mediate interactions with the host. Functions identified in this work could be potential targets for preventive and therapeutic anti-enterococcal regimens.

## Materials and Methods

### Bacterial strains, plasmids and culture conditions

*Enterococcus faecalis* V583 is a vancomycin resistant strain [25] harboring three plasmids. Nucleotide sequences of the chromosome and plasmids are available at http://www.tigr.org. For technical reasons we conducted the study with a plasmid cured strain. Plasmid curing was carried out by a procedure combining three conditions known to lead to plasmid loss: the use of novobiocin [77] and growth at 42°C in a medium lacking its buffering component [78]. Strain V583 was cultured for 96 hours under these conditions and the procedure was repeated several times. The successive strains obtained were: V583 EryS (carrying pTEF2, and pTEF1 free of the EryR cassette), V32 (containing pTEF1 free of the EryR cassette), and V19, renamed in this study VE14089 (free of replicating plasmids). A transcriptional microarray was performed on strain VE14089 using the Affymetrix GeneChip, SLARE1 [79], the details of transcriptome experiments and analysis are described as additional information in Text S1. Microarray data have been deposited in the ArrayExpress public database at http://www.ebi.ac.uk/ arrayexpress with the series accession number E-MEXP-3068. The microarray analysis revealed that genes *efA0063* to *efA0006* from plasmid pTEF1 were still present in this strain but PCR analysis at the borders of this region demonstrated that a circular form of the plasmid was not present. To identify the site of chromosomal integration, pulsed-field gel electrophoresis of *Not*I, *SfiI*, or *Sma*I digested VE14089 genomic DNA and subsequent Southern hybridization was performed as described previously [80]. An intragenic probe for *efA0078* was amplified using the primers, efA78F 5′-GGACTTGATCCATTGAAGAC-3′, and efA78R 5′-GGTAAAACCTGACTATTGCC-3′, and radiolabeled by incorporation of [α-^32^P]dCTP-labelled deoxynucleotides (Perkin Elmer) using Ready-To-Go DNA labelling beads (GE). Southern blot analysis revealed a chromosomally integrated plasmid-fragment on two possible *Sma*I restriction fragments of 105 kb or 110 kb. To determine the site of integration, long-range PCRs were performed in the 110-kb region, which allowed delimiting the insertion within an intergenic region between the genes *ef3209* and *ef3210*. Finally, the precise integration site of the genes *efA0063* to *efA0006* was determined by sequencing.

*E. coli* was cultivated at 37°C in LB broth (Difco) in a glass tube with shaking. *E. faecalis* strains were grown in M17 broth (Difco) supplemented with 0.5% glucose (GM17), or on GM17 agar plates at an appropriate temperature. *E. coli* VE14188 (*dam* ^−^ *dcm* ^−^ RepA^+^) was used to amplify plasmids to be introduced in *E. faecalis*. To maintain the conditionally replicating plasmid pVE14218 and derivative plasmids, tetracycline was used at 12.5 µg/mL for *E. coli* and at 10 µg/mL for *E. faecalis*. To maintain plasmid pGhost3 (RepA^Ts^), chloramphenicol was used at 10 µg/mL.

### Transformation of *E. coli* and of *E. faecalis*

*E. coli* VE14188 and *E. faecalis* were transformed by electroporation using a BioRad Gene Pulser apparatus according to the method of Dower et al. [81] and Holo and Nes [82], respectively. Briefly, 40 µL of electrocompetent cells of *E. faecalis* VE14412 were mixed with 2 µL of plasmid DNA (100 ng). After electric shock, 1 mL of SM17MC medium (GM17 with 0,5 M saccharose, 10 mM MgCl_2_ and 10 mM CaCl_2_, pH 6,8–7,0) were added and the cell suspension was incubated at 28°C for 2 h without shaking. One hundred µL of 10^0^ and 10^−1^ dilutions were spread on GM17 agar plates with 10 µg/mL tetracycline and incubated at 28°C for 48 h.

### Extraction of plasmid DNA from *E. coli* and of total DNA from *E. faecalis*

DNA of pVE14218 was purified using cesium chloride gradient centrifugation [83] and cognate derivatives plasmids were purified according to the protocol of the QIAprep Spin Miniprep Kit (QIAGEN).

Total DNA from *E. faecalis* was extracted using a modified protocol described by Brinster et al., 2007 [84]. Bacterial cells were pelleted by centrifugation at 4°C, 8,000 rpm for 3 min. The cell pellet was washed once in 1 ml of TES (50 mM Tris, pH 7.5; 20% sucrose, 5 mM EDTA), resuspended in 300 µL of TES+lysozyme (5 mg/mL) and incubated for 30 min at 37°C. Protoplasts were mixed with 300 µL of saline solution (150 mM NaCl; 10 mM EDTA, pH 8) and lysed by the addition of 20 µL of 20% SDS. One volume of phenol/chloroform/isoamyl alcohol 25∶24∶1 (PCI) was added and the suspension was homogenised. DNA was separated from cellular debris by centrifugation for 10 min at 14,000 rpm at 10°C. The PCI step was repeated once or twice on the aqueous phase containing soluble DNA. Subsequently, the DNA was precipitated by addition of one volume of isopropanol. After centrifugation at 14,000 rpm, 10°C for 10 min, the supernatant was discarded, the DNA pellet was washed in cold 70% ethanol, centrifuged for 5 min and then air-dried. DNA was finally resuspended in 50 µL of TE+RNAse (100 µg/mL), incubated at least 30 min at 37°C. and stored at −20°C.

### Generation of integrants of *E. faecalis* by thermal shock

Single colonies of *E. faecalis* containing both pGhost3, which provides functional RepA^Ts^, and the conditionally replicating pVE14218-derivative were cultivated in 50 ml of GM17 (without antibiotic) under static conditions for 2 h at 28°C. Then, the cultures were transferred to the non-permissive temperature for pGhost3 (42°C) for two hours to inhibit plasmid replication. Next, serial dilutions were plated on GM17 containing 10 µg/ml tetracycline (to select for plasmid integrants) and on GM17 (to evaluate the integration rate) and incubated for 24 h at 42°C. Thus, chromosomal integration of the specific pVE14218 derivative and loss of pGhost3 was selected for. Integration at the targeted locus was validated by Southern Blot analysis using the DIG DNA labeling and Detection kit (Roche) and/or PCR.

### H_2_O_2_ stress resistance assay

Two ml of an overnight culture of *E. faecalis* was harvested, and cells were suspended in 10 ml of GM17 medium. A volume of 180 µl of the resuspended culture was added to each well of a microtiter plate to which 20 µl of 0 mM, 5 mM, 6 mM or 7 mM H_2_O_2_ was added (thus giving 1/10^th^ final concentration; plates were incubated for 2 h at 37°C without agitation. Twenty µl of culture from each well was then used to inoculate another microtiter plate containing in each well 180 µl of GM17. Plates were incubated at 37°C in a plate reader (BioRad Model 680), and growth was monitored for 24 h by for OD_600_ every 30 min after plate agitation for 10 s.

### Antibiotic resistance assay

Resistance to 34 antibiotics, covering different classes. Both intrinsic and acquired resistances were assessed, at first using disk susceptibility tests. Screens were done according to the recommendations of the disk providers. Subsequently, the minimal inhibitory concentration (MIC) was determined for each antibiotic using either the microdilution, agar dilution or E-test method and results were interpreted according to the recommendations of the Clinical and Laboratory Standards Institute (CLSI, formerly NCCLS). The MICs were determined for mutants showing relevant antibiotics phenotypes by the disk diffusion assays. MIC values of mutant that were at least 2-fold different from that of the parent strain VE14089 were considered significant.

### Resistance to opsonophagocytosis

Rabbit sera raised against whole bacterial cells and known to contain opsonic antibodies against *E. faecalis* V583 were tested for their opsonic killing activity against *E. faecalis* reference strains as described previously [31]. Polymorphonuclear neutrophils (PMN) were freshly prepared from human blood collected from healthy adult volunteers and percent killing was calculated by comparing the colony counts in the inoculum (i.e., viable counts at time 0 – T0) to the colony counts after a 90-minute incubation at 37°C (T90), using the following formula: ((mean cfu at T0 – mean cfu at T90)/(mean cfu at T0))×100. The killing activity of sera in combination with complement and PMN was compared to control samples with human sera and complement but without PMN. While the VE14089 strain was killed only by a combination of PMNs, complement, and antibodies, we observed the following differences in some of the mutants: Killing by complement alone (Complement Sensitivity: Co-Sens), Killing by complement and PMNs but without antiserum (Serum Independent: Ser-Ind), and a general higher resistance to killing with all three components (Higher Resistance, High-Res).

### Adhesion of *E. faecalis* to Caco-2 cells

The Caco-2 cell line from human colon adenocarcinoma was obtained from the cell bank of the Centro de Investigaciones Biologicas (CIB-CSIC, Madrid, Spain). Caco-2 cells were cultured at 37°C under 5% CO_2_ in 24-well plastic microplates (TTP) in RPMI medium 1640 (Invitrogen) containing 10% Foetal Bovine Serum (Invitrogen), without antibiotics, until the cells were grown to ±95% confluence. The cell monolayers were washed with sterile Phosphate Buffered Saline without Ca^2+^ and Mg^2+^ (Biowhitaker). The RPMI medium was removed, and fresh RPMI medium was added before infection. The bacterial inoculum consisted of a suspension in RPMI without Foetal Bovine Serum of bacteria obtained from 1 ml (∼10^9^ bacteria) of an overnight culture in 5 ml BHI at 37°C, centrifuged during 5 min at 6000 rpm. Briefly, bacterial suspensions were added to ±95% confluent Caco-2 monolayers at a multiplicity of infection (MOI) of 50∶1. Infected cell monolayers were centrifuged for 5 min at 500 rpm at room temperature and incubated for 2 h at 37°C under 5% CO_2_. They were then washed 3 times with PBS. A 300-µl volume of triton X-100 (Sigma) in 1% PBS was added per well. Homogenates were collected and serial dilutions were plated on BHI containing 10 µg/µl tetracycline for mutants and on BHI for controls (strains V583 and VE14089) and incubated for 24 h at 37°C. Colonies were counted to assess adherent/invasive colony forming units (CFU). Experiments were performed three times, in duplicate.

### Galleria mellonella killing assay

*E. faecalis* mutants were grown in GM17 containing 1 µg/ml of tetracycline and collected by centrifugation 1 h after they had reached the stationary phase. The VE14089 strain was grown in GM17. Bacterial cells were washed twice with 0.9% saline solution and stored as a dry frozen pellet at −80°C. At least two days before inoculation, the frozen bacterial pellet was suspended in 1 ml of saline solution and serial dilutions were plated on GM17 agar plates in order to determine the bacterial count of the pellet.

Groups of 30 last instar *G. mellonella* larvae, weighing about 200 mg each, were injected at the base of the last proleg with 10 µl of a bacterial inoculum (∼4×10^8^ cells per ml) using a microinjector (KDS 100, KD Scientific Inc.) with a 1-ml syringe and 0.45- by 12-mm needles (Terumo). A control group of larvae received 10 µl of a saline solution only. The inoculum size was confirmed by determining the number of CFU on GM17 agar. The infected larvae were kept, five per Petri dish, at 37°C and survival was monitored every 24 h for 2 to 5 days depending on the experiment. Survival obtained with mutant strains might be overestimated if gene reversion occurs, although in view of the short time of the assays, we consider this an unlikely event. Survival curves were constructed by the Kaplan-Meier method and compared by log-rank analysis (GraphPad Prism, version 4.0; GraphPad Software, Inc.). Whereas *P* values of >0.05 were considered not significantly different from the VE14089 (nsd), *P* values of <0.05 were considered statistically significant with the following classification: 0.005<P<0.05 (decreased or increased), 0.0005<P<0.005 (decreased+ or increased+) and P<0.0005 (decreased++ or increased++).

### Phenotypic data base construction

Information on the mutant construction (primers and plasmids used) and on the results of the screening was added in the database FAECALVIR provided by Migale (INRA, Jouy-en-Josas, France) and implemented by all the partners of the study (http://genome.jouy.inra.fr/phenotype/).

### Ethics statement

The animal welfare committees of the University of Freiburg (Regierungspräsidium Freiburg Az 35/9185.81/G-07/15) approved animal experiments. Rabbit experiments were performed in compliance with the German animal protection law (TierSchG). The animals were housed and handled in accordance with good animal practice as defined by FELASA and the national animal welfare body GV-SOLAS.

Human PMNs were obtained from healthy volunteers in accordance with a protocol approved by University of Freiburg Institutional Review Board for Human Subjects (approval number 116/04). Written informed consent was obtained from all human volunteers.

## Supporting Information

Figure S1 **Comparison of growth curves of strains** ***E. faecalis*** **V583 (○) and VE14089 (□) grown aerobically (A) or anaerobically (B) in GM17 medium.** The kinetics of growth was monitored at OD600 nm. Mean values of two independent experiments are shown. (C) Survival of *Galleria mellonella* after infection by *E. faecalis* V583 (○) and VE14089 (□).
(PDF)

Figure S2 **Survival of** ***Galleria mellonella*** **after injection of** ***E. faecalis*** **single cross-over mutant strains (SCO) compared to VE14089 strain.** Representative survival rates are shown and correspond to the phenotype not significant (A), decreased (B), decreased+ (C), decreased++ (D), increased (E) and increased++ (F).
(PDF)

Table S1 **Genes mutated in this study, their place in the JCVI role categories and phenotypes of the corresponding** ***E. faecalis*** **mutants.**
(DOC)

Table S2 **Insertionally inactivated genes in mutants affected in oxidative stress response.**
(DOC)

Table S3 **Insertionally inactivated genes in mutants affected in antibiotic resistance.**
(DOC)

Table S4 **Insertionally inactivated genes in mutants affected in resistance to opsonophagocytosis test.**
(DOC)

Table S5 **Insertionally inactivated genes in mutants affected in the adhesion to Caco-2 cells.**
(DOC)

Table S6 **Insertionally inactivated genes in mutants affected in virulence towards** ***Galleria mellonella*** **.**
(DOC)

Text S1 **Supplementary text describing additional materials and methods utilized in the microarray transcriptomic experiment and list of antibiotics used in this study.**
(DOC)
