# Supplementary figures and images for: Large-Scale Screening of a Targeted Enterococcus faecalis Mutant Library Identifies Envelope Fitness Factors
Source: PLoS One. 2011 Dec 15;6(12):e29023. doi: 10.1371/journal.pone.0029023 (PMC3240637; doi:10.1371/journal.pone.0029023)

Figure S1

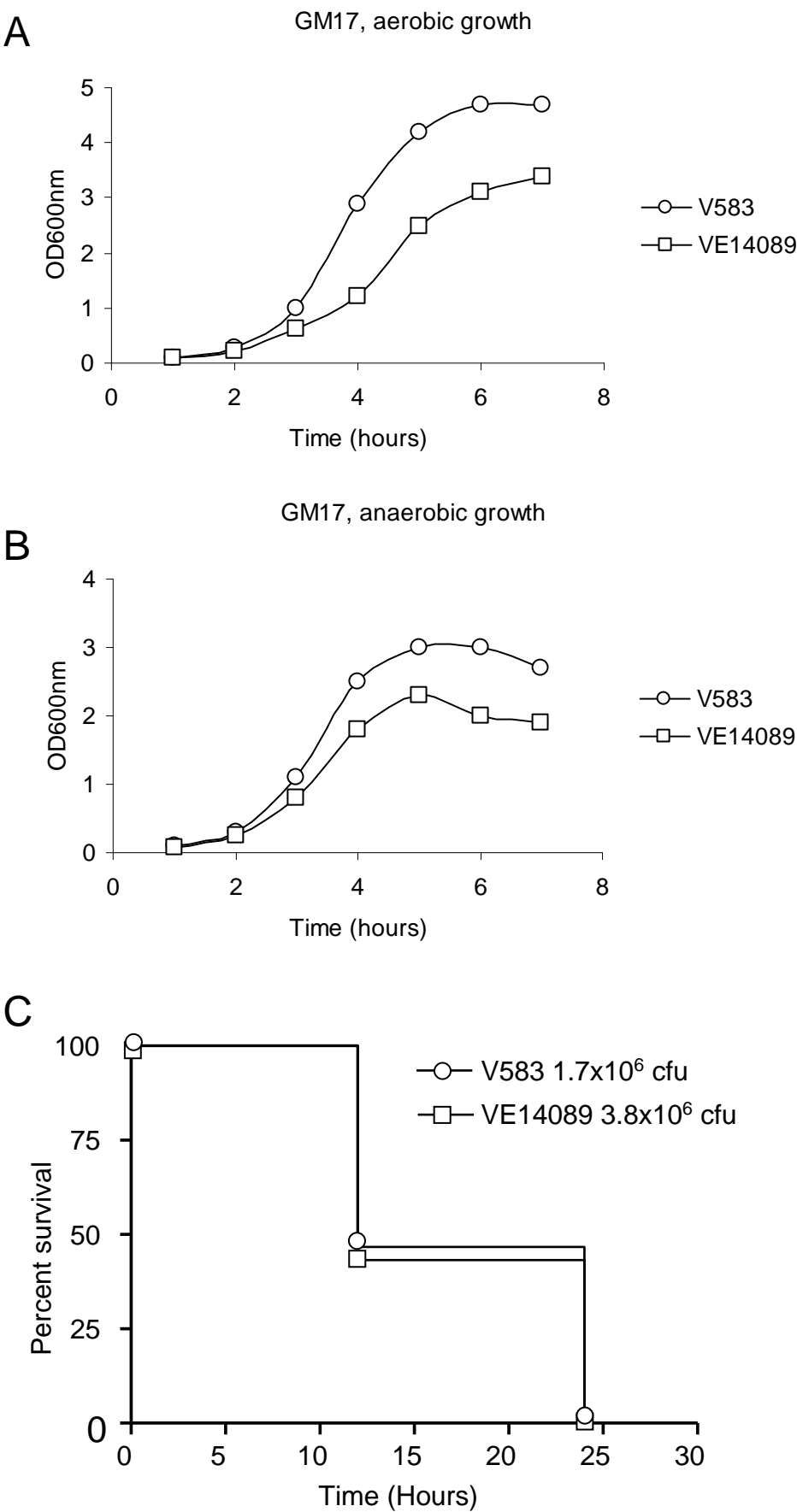

Supplement: Figure S1 — Comparison of growth curves of strains E. faecalis V583 (○) and VE14089 (□) grown aerobically (A) or anaerobically (B) in GM17 medium. The kinetics of growth was monitored at OD600 nm. Mean values of two independent experiments are shown. (C) Survival of Galleria mellonella after infection by E. faecalis V583 (○) and VE14089 (□). (PDF) [file pone.0029023.s001.pdf]

Figure S2

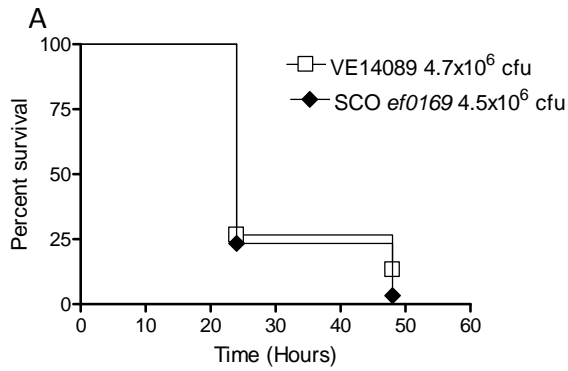

Not significant ( $P > 0.05$ )

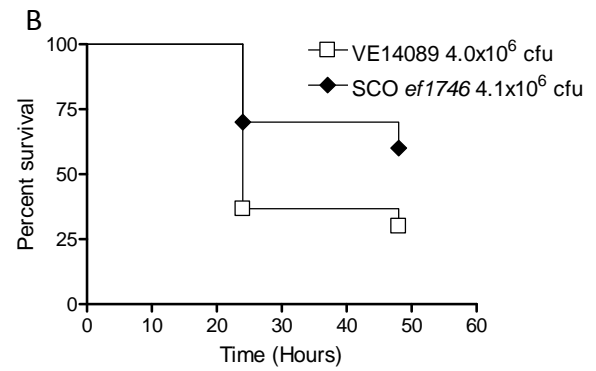

Decreased ( $P < 0.05$ )

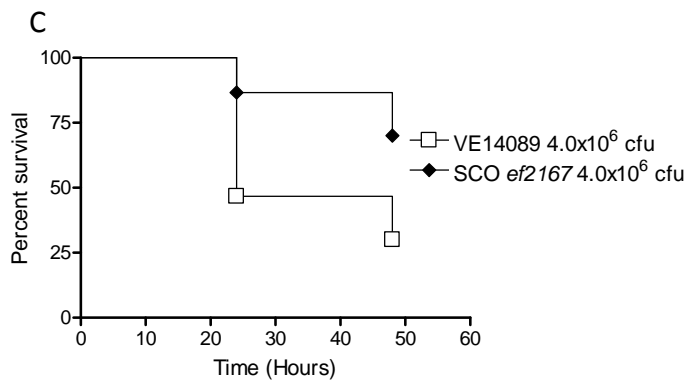

Decreased + ( $P < 0.005$ )

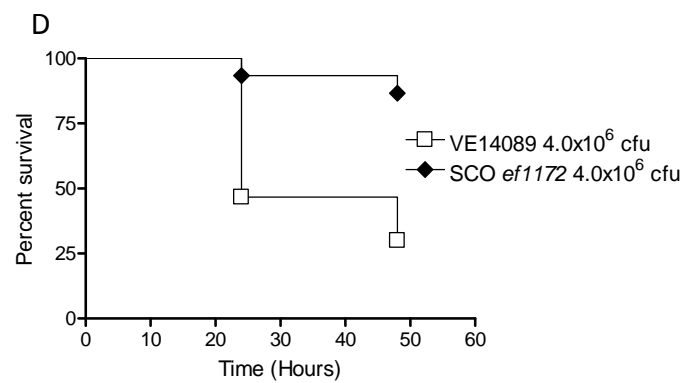

Decreased ++ ( $P < 0.0005$ )

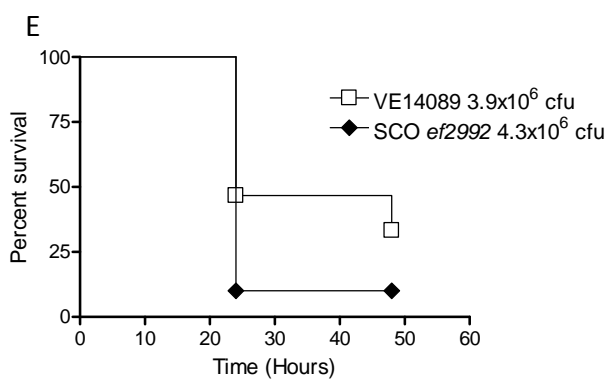

Increased ( $P < 0.05$ )

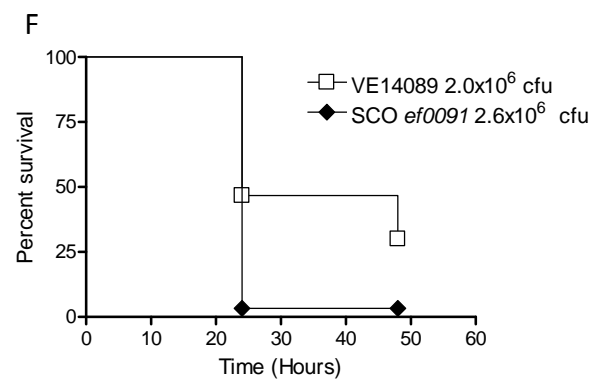

Increased++ ( $P < 0.0005$ )

Supplement: Figure S2 — Survival of Galleria mellonella after injection of E. faecalis single cross-over mutant strains (SCO) compared to VE14089 strain. Representative survival rates are shown and correspond to the phenotype not significant (A), decreased (B), decreased+ (C), decreased++ (D), increased (E) and increased++ (F). (PDF) [file pone.0029023.s002.pdf]
