# Supplementary material for: Large-Scale Screening of a Targeted Enterococcus faecalis Mutant Library Identifies Envelope Fitness Factors
Source: PLoS One. 2011 Dec 15;6(12):e29023. doi: 10.1371/journal.pone.0029023 (PMC3240637; doi:10.1371/journal.pone.0029023)
Supplement: Table S1 — Genes mutated in this study, their place in the JCVI role categories and phenotypes of the corresponding E. faecalis mutants. (DOC) [file pone.0029023.s003.doc]

**Table S1.** List of the mutated genes, JCVI role categories and results obtained with the phenotypes.

| Locus | Protein function | JCVI role category | Sensitivity to H_2_O_2_^a^ | Sensitivity to Antibiotic^b^ | Opsono- assay^c^ | Adhesion to CaCo2 | Virulence in  *G. mellonella*^d^ | Growth ability |
| --- | --- | --- | --- | --- | --- | --- | --- | --- |
| EF0031 | membrane protein, putative | Cell envelope | - | - | - | - | - | - |
| EF0055 | adhesion lipoprotein | Cellular processes | - | - | - | - | - | - |
| EF0071 | lipoprotein, putative | Cell envelope | +++ | - | - | - | - | - |
| EF0073 | transcriptional regulator, Cro/CI family | Regulatory functions | +++ | - | - | - | Decreased+ | - |
| EF0079 | gls24 protein | Cellular processes | ++ | - | - | - | - | - |
| EF0080 | gls24 protein | Cellular processes | ++ | - | - | Increased | - | - |
| EF0082 | major facilitator family transporter | Transport and binding proteins | - | - | - | - | - | - |
| EF0086 | conserved domain protein | Hypothetical proteins | - | - | - | Increased | Decreased | - |
| EF0089 | conserved domain protein | Hypothetical proteins | - | - | - | - | - | - |
| EF0091 | conserved hypothetical protein | Hypothetical proteins | - | - | - | - | Increased++ | - |
| EF0100 | seryl-tRNA synthetase | Protein synthesis | - | - | - | - | - | - |
| EF0107 | transcriptional regulator, Crp/Fnr family | Regulatory functions | +++ | - | - | - | - | - |
| EF0146 | surface exclusion protein, putative | Cellular processes | ++ | AmoxRes | - | - | - | - |
| EF0169 | lipase/acylhydrolase | Fatty acid and phospholipid metabolism | - | - | - | - | - | - |
| EF0176 | basic membrane protein family | Cell envelope | - | - | Ser-Ind | - | - | Decreased |
| EF0177 | basic membrane protein family | Cell envelope | - | - | - | - | - | - |
| EF0201 | translation elongation factor Tu | Protein synthesis | +++ | - | - | Increased | - | - |
| EF0252 | N-acetylmuramoyl-L-alanine amidase, family 4 | Cell envelope | - | - | - | Decreased | Decreased++ | - |
| EF0280 | cation efflux family protein | Transport and binding proteins | - | - | - | - | - | - |
| EF0362 | chitin binding protein, putative | Cell envelope | - | - | - | - | Decreased++ | - |
| EF0384 | hypothetical protein | No Data | - | - | - | - | - | - |
| EF0386 | carbamate kinase | Energy metabolism | - | - | - | - | - | - |
| EF0389 | membrane protein, putative | Cell envelope | - | AmoxRes | - | - | - | - |
| EF0392 | hypothetical protein | No Data | ++ | - | - | - | - | - |
| EF0403 | transcriptional regulator, MarR family | Regulatory functions | - | - | - | - | Decreased | - |
| EF0420 | drug resistance transporter, EmrB/QacA family protein | Transport and binding proteins | - | - | - | - | - | - |
| EF0465 | transcriptional regulator | Regulatory functions | ++ | - | - | - | Decreased | - |
| EF0502 | membrane protein, putative | Cell envelope | - | - | - | - | - | - |
| EF0516 | membrane protein, putative | Cell envelope | - | AmoxRes | - | - | - | - |
| EF0541 | PTS system component, authentic frameshift | Signal transduction | - | AmoxRes | - | - | Decreased+ | - |
| EF0553 | PTS system, IID component | Signal transduction | - | AmoxRes | - | Decreased | - | - |
| EF0559 | polysaccharide biosynthesis family protein | Cell envelope | - | - | - | - | - | - |
| EF0567 | potassium-transporting ATPase, subunit A | Transport and binding proteins | - | - | - | - | - | - |
| EF0573 | hypothetical protein | No Data | +++ | - | - | - | - | - |
| EF0577 | adhesion lipoprotein | Cellular processes | - | - | - | Increased | - | - |
| EF0579 | transcriptional regulator, putative | Regulatory functions | - | - | - | Increased | Decreased++ | - |
| EF0583 | ABC transporter, ATP-binding protein/permease protein | Transport and binding proteins | - | - | - | - | Increased+ | - |
| EF0590 | polysaccharide deacetylase family protein | Energy metabolism | ++ | - | - | - | - | - |
| EF0600 | transcriptional regulator, TetR family | Regulatory functions | - | - | High-Res | - | Decreased++ | - |
| EF0601 | transcriptional regulator, TetR family | Regulatory functions | - | - | High-Res | - | Decreased++ | - |
| EF0604 | gls24 protein | Cellular processes | +++ | - | - | - | - | - |
| EF0609 | conserved hypothetical protein | Hypothetical proteins | - | - | - | Increased | - | - |
| Locus | Protein function | JCVI role category | Sensitivity to H_2_O_2_^a^ | Sensitivity to Antibiotic^b^ | Opsono- assay^c^ | Adhesion to CaCo2 | Virulence in  *G. mellonella*^d^ | Growth ability |
| EF0617 | membrane protein, putative | Cell envelope | - | - | - | Increased | - | Decreased |
| EF0644 | transcriptional regulator, LysR family | Regulatory functions | - | - | - | - | - | - |
| EF0746 | penicillin-binding protein, putative | Cell envelope | ++ | - | - | Increased | - | - |
| EF0779 | glycerophosphoryl diester phosphodiesterase family protein | Fatty acid and phospholipid metabolism | - | - | - | - | - | - |
| EF0785 | drug resistance transporter, EmrB/QacA family protein | Transport and binding proteins | - | - | - | - | Decreased+ | - |
| EF0786 | tributyrin esterase, putative | Fatty acid and phospholipid metabolism | - | - | - | - | - | - |
| EF0797 | conserved domain protein | Hypothetical proteins | - | - | - | - | - | - |
| EF0814 | transcriptional regulator, GntR family | Regulatory functions | + | - | - | Increased | Decreased++ | - |
| EF0818 | polysaccharide lyase, family 8 | Cellular processes | - | CefSen | - | - | Decreased | - |
| EF0876 | hypothetical protein, MGA Helix-turn-helix domain | No Data | - | - | - | Decreased | Decreased++ | - |
| EF0887 | glycosyl transferase, group 2 family protein | Cell envelope | - | - | - | - | Decreased++ | Decreased |
| EF0889 | conserved hypothetical protein | Hypothetical proteins | - | - | - | - | - | - |
| EF0892 | amino acid ABC transporter, ATP-binding protein | Transport and binding proteins | - | - | - | - | - | - |
| EF0906 | conserved hypothetical protein | Hypothetical proteins | - | - | - | - | Increased++ | - |
| EF0929 | amino acid permease family protein | Transport and binding proteins | - | - | - | - | - | - |
| EF0962 | transcriptional regulator, AraC family | Regulatory functions | - | - | - | - | - | - |
| EF0994 | UDP-N-acetylglucosamine--N-acetylmuramyl-(pentapeptide) pyrophosphoryl-undecaprenol N-acetylglucosamine transferase | Cell envelope | - | - | - | Increased | Decreased+ | - |
| EF1027 | membrane protein, putative | Cell envelope | + | - | Ser-Ind | Increased | Decreased++ | - |
| EF1042 | multidrug resistance protein, putative | Transport and binding proteins | - | - | - | - | - | - |
| EF1076 | streptomycin 3-adenylyltransferase, putative | Cellular processes | - | - | - | Decreased | - | - |
| EF1100 | ABC transporter, ATP-binding/permease protein | Transport and binding proteins | - | - | - | - | - | - |
| EF1156 | transcriptional regulator, GntR family | Regulatory functions | - | - | - | - | - | - |
| EF1157 | peptidase, M20/M25/M40 family | Protein fate | - | - | - | - | - | - |
| EF1172 | teichoic acid biosynthesis protein B, putative | Cell envelope | +++ | - | Ser-Ind | Increased | Decreased++ | Decreased |
| EF1173 | glycosyl transferase, WecB/TagA/CpsF family | Cell envelope | +++ | - | Ser-Ind | - | - | Decreased |
| EF1175 | glycerol-3-phosphate cytidylyltransferase | Cell envelope | - | - | - | - | Decreased++ | - |
| EF1193 | DNA-binding response regulator VicR | Signal transduction | - | ChloramRes | - | - | - | - |
| EF1211 | NADH peroxidase | Energy metabolism | - | CeftriSen++ | - | - | Decreased++ | - |
| EF1212 | transcriptional regulator | Regulatory functions | + | - | - | - | Decreased | - |
| EF1224 | transcriptional regulator, Cro/CI family | Regulatory functions | - | - | - | - | - | - |
| EF1238 | glycosyl hydrolase, family 3 | Energy metabolism | +++ | - | - | - | - | - |
| EF1288 | conserved hypothetical protein | Hypothetical proteins | + | - | - | - | - | - |
| EF1347 | glycosyl hydrolase, family 13 | Energy metabolism | ++ | pleiotrophic effects | - | - | - | - |
| EF1408 | ABC transporter, ATP-binding protein | Transport and binding proteins | +++ | - | - | - | - | - |
| EF1413 | msrC protein, putative | Cellular processes | - | - | - | - | - | - |
| EF1420 | hypothetical protein | No Data | ++ | - | - | - | Decreased++ | - |
| EF1493 | V-type ATPase, subunit I | Transport and binding proteins | ++ | - | - | - | Decreased | - |
| EF1516 | PTS system, IIABC components | Signal transduction | - | - | - | Increased | - | - |
| EF1525 | transcriptional regulator, Fur family | Regulatory functions | +++ | GenRes | - | Increased | - | - |
| EF1583 | N-acetylmuramoyl-L-alanine amidase, family 4 | Cell envelope | ++ | - | - | - | - | - |
| EF1585 | transcriptional regulator, Fur family | Regulatory functions | - | - | - | - | Decreased++ | - |
| EF1592 | ABC transporter, ATP-binding/permease protein | Transport and binding proteins | - | - | - | - | Decreased+ | - |
| EF1597 | catalase/peroxidase | Cellular processes | - | - | - | - | - | - |
| Locus | Protein function | JCVI role category | Sensitivity to H_2_O_2_^a^ | Sensitivity to Antibiotic^b^ | Opsono- assay^c^ | Adhesion to CaCo2 | Virulence in  *G. mellonella*^d^ | Growth ability |
| EF1599 | TPR domain transcriptional regulator, Cro/CI family | Regulatory functions | +++ | - | - | - | - | - |
| EF1606 | glycosyl hydrolase, family 1 | Energy metabolism | - | - | - | Increased | - | - |
| EF1656 | transcriptional regulator, LysR family | Regulatory functions | - | - | - | - | - | - |
| EF1663 | branched-chain phosphotransacylase | Fatty acid and phospholipid metabolism | - | - | - | - | Decreased | - |
| EF1681 | peptide methionine sulfoxide reductase | Protein fate | + | - | - | - | - | - |
| EF1705 | phosphate-binding protein | Transport and binding proteins | +++ | - | Ser-Ind | Increased | Decreased | - |
| EF1709 | sugar-binding transcriptional regulator, GntR family | Regulatory functions | - | - | - | - | - | - |
| EF1741 | catabolite control protein A | Regulatory functions | ++ | - | - | - | Decreased++ | - |
| EF1743 | proline dipeptidase | Protein fate | - | - | - | - | - | - |
| EF1746 | UTP-glucose-1-phosphate uridylyltransferase | Cell envelope | +++ | FusidASen | Ser-Ind | - | Decreased | - |
| EF1759 | phosphate ABC transporter, phosphate-binding protein | Transport and binding proteins | - | GenRes | - | - | Decreased+ | - |
| EF1760 | cell division ABC transporter, permease protein FtsX, putative | Transport and binding proteins | + | - | - | - | Decreased | - |
| EF1798 | hypothetical protein | No Data | +++ | - | - | - | Decreased | - |
| EF1814 | drug resistance transporter, EmrB/QacA family protein | Transport and binding proteins | - | - | - | - | Decreased++ | - |
| EF1824 | glycosyl hydrolase, family 31/fibronectin type III domain protein | Cell envelope | - | - | - | - | - | - |
| EF1851 | glycosyl hydrolase, family 35 | Energy metabolism | - | - | - | - | Increased++ | - |
| EF1864 | DNA-binding response regulator | Signal transduction | - | - | - | - | Decreased++ | - |
| EF1869 | permease, putative | Transport and binding proteins | ++ | - | - | Increased | - | - |
| EF1904 | glycerophosphoryl diester phosphodiesterase family protein | Fatty acid and phospholipid metabolism | - | - | - | - | - | - |
| EF1920 | C4-dicarboxylate anaerobic carrier | Transport and binding proteins | ++ | - | - | - | - | - |
| EF1943 | drug resistance transporter, Bcr/CflA family protein | Transport and binding proteins | - | - | - | - | - | - |
| EF2020 | hypothetical protein | No Data | +++ | - | - | - | - | - |
| EF2068 | multidrug resistance protein, putative | Transport and binding proteins | - | - | - | - | Decreased++ | - |
| EF2076 | endocarditis specific antigen | Cellular processes | - | - | - | - | - | - |
| EF2144 | lipoprotein, putative | Cell envelope | - | - | - | - | - | - |
| EF2163 | glycerophosphoryl diester phosphodiesterase, putative | Fatty acid and phospholipid metabolism | - | - | - | - | - | - |
| EF2167 | glycosyl transferase, group 2 family protein | Cell envelope | - | MetSen | Ser-Ind | Increased | Decreased+ | Decreased |
| EF2170 | glycosyl transferase, group 2 family protein | Cell envelope | +++ | - | Ser-Ind | - | Decreased++ | Decreased |
| EF2180 | glycosyl transferase, group 2 family protein | Cell envelope | +++ | - | - | - | - | - |
| EF2181 | glycosyl transferase, group 2 family protein | Cell envelope | - | FusidASen | - | - | Decreased++ | - |
| EF2190 | glycosyl transferase, group 2 family protein | Cell envelope | - | - | - | - | - | Decreased |
| EF2195 | glycosyl transferase, group 2 family protein | Cell envelope | + | - | - | - | - | - |
| EF2196 | glycosyl transferase, group 2 family protein | Cell envelope | +++ | - | Ser-Ind | Increased | Decreased++ | Decreased |
| EF2197 | glycosyl transferase, group 2 family protein | Cell envelope | - | GenRes | - | - | Decreased++ | Increased |
| EF2198 | glycosyl transferase, group 4 family protein | Cell envelope | +++ | - | Ser-Ind | - | Decreased++ | Decreased |
| EF2221 | ABC transporter, substrate-binding protein | Transport and binding proteins | + | - | - | - | - | - |
| EF2248 | hypothetical protein | No Data | - | - | - | - | - | - |
| EF2250 | conserved domain protein | Hypothetical proteins | - | - | - | - | Decreased++ | - |
| EF2268 | conserved hypothetical protein | Hypothetical proteins | + | - | - | - | - | - |
| EF2276 | hypothetical protein | No Data | - | - | - | - | Increased | - |
| EF2318 | peptidase, M23/M37 family | Protein fate | - | - | - | - | - | - |
| EF2347 | cell wall surface anchor family protein | Cell envelope | ++ | - | - | - | - | - |
| EF2417 | transcriptional regulator, Fur family | Regulatory functions | - | - | - | - | Decreased++ | - |
| EF2425 | phosphoglucomutase/phosphomannomutase family protein | Energy metabolism | - | - | - | - | - | - |
| Locus | Protein function | JCVI role category | Sensitivity to H_2_O_2_^a^ | Sensitivity to Antibiotic^b^ | Opsono- assay^c^ | Adhesion to CaCo2 | Virulence in  *G. mellonella*^d^ | Growth ability |
| EF2442 | phosphate transporter family protein | Transport and binding proteins | ++ | - | - | - | Decreased++ | - |
| EF2464 | conserved domain protein | Hypothetical proteins | - | - | - | - | - | - |
| EF2490 | conserved hypothetical protein | Hypothetical proteins | + | - | High-Res | - | - | - |
| EF2491 | glycosyl transferase, group 2 family protein | Cell envelope | - | - | - | - | - | - |
| EF2492 | glycosyl transferase, group 2 family protein | Cell envelope | - | - | - | - | - | - |
| EF2505 | cell wall surface anchor family protein | Cell envelope | - | - | - | - | - | - |
| EF2525 | cell wall surface anchor family protein | Cell envelope | - | - | - | Increased | - | - |
| EF2556 | fumarate reductase flavoprotein subunit precursor, putative | Energy metabolism | - | - | - | Increased | - | - |
| EF2618 | carboxylesterase precursor, putative | Fatty acid and phospholipid metabolism | - | - | - | - | - | - |
| EF2662 | choline binding protein | Cellular processes | - | - | - | - | Decreased | - |
| EF2671 | conserved hypothetical protein | Hypothetical proteins | ++ | - | - | - | - | - |
| EF2682 | conserved hypothetical protein | Hypothetical proteins | +++ | - | - | - | - | - |
| EF2703 | transcriptional regulator | Regulatory functions | - | - | - | - | - | - |
| EF2709 | glycosyl hydrolase, family 2 | Energy metabolism | - | - | - | - | - | - |
| EF2746 | dltD protein | Cell envelope | - | - | - | - | Decreased++ | - |
| EF2748 | peptidase, U32 family, putative | Protein fate | - | - | - | - | Decreased++ | - |
| EF2750 | conserved hypothetical protein | Hypothetical proteins | - | - | - | - | - | - |
| EF2772 | drug resistance transporter, putative, authentic frameshift | Transport and binding proteins | - | - | - | Increased | - | - |
| EF2773 | major facilitator family transporter | Transport and binding proteins | - | - | - | - | - | - |
| EF2795 | LysM domain lipoprotein | Cell envelope | - | - | - | - | - | - |
| EF2796 | hypothetical protein | No Data | - | GenRes | - | Increased | - | - |
| EF2889 | 2-hydroxy-3-oxopropionate reductase | Energy metabolism | - | CefSen | - | - | - | - |
| EF2890 | glycosyl transferase, group 1 family protein | Cell envelope | - | GenRes | Co-Sens | - | - | - |
| EF2891 | glycosyl transferase, group 1 family protein | Cell envelope | - | MetRes | - | - | - | - |
| EF2958 | transcriptional regulator, LysR family | Regulatory functions | - | - | - | - | - | - |
| EF2963 | esterase, putative | Unknown function | - | - | - | - | - | - |
| EF2932 | AhpC/TSA family protein | Unknown function | - | - | - | - | - | - |
| EF2992 | major facilitator family transporter | Transport and binding proteins | +++ | GenRes | - | Increased | Increased | - |
| EF2997 | peptidase, M20/M25/M40 family | Protein fate | - | ChloramRes | - | - | Decreased+ | - |
| EF3012 | membrane protein, putative | Cell envelope | - | - | - | - | - | - |
| EF3023 | polysaccharide lyase, family 8 | Cellular processes | - | - | - | - | - | - |
| EF3034 | transcriptional regulator, GntR family | Regulatory functions | - | - | - | - | Increased | - |
| EF3041 | pheromone binding protein | Transport and binding proteins | - | - | - | - | - | - |
| EF3060 | secreted lipase, putative | Unknown function | + | - | - | - | - | - |
| EF3103 | membrane protein, putative | Cell envelope | - | - | - | - | - | - |
| EF3156 | transcriptional regulator, GntR family | Regulatory functions | - | - | - | - | - | - |
| EF3157 | glycosyl hydrolase, family 65 | Energy metabolism | - | - | - | - | Decreased++ | - |
| EF3164 | PilB family protein | Unknown function | +++ | - | - | - | - | - |
| EF3180 | RNA polymerase sigma-70 factor, ECF subfamily | Transcription | - | - | - | - | - | - |
| EF3191 | lipase, putative | Fatty acid and phospholipid metabolism | - | - | - | - | - | - |
| EF3206 | adhesion lipoprotein | Cellular processes | - | - | - | - | - | - |
| EF3245 | cell-envelope associated acid phosphatase | Unknown function | - | - | - | - | - | - |
| EF3255 | thiamin biosynthesis lipoprotein ApbE, putative | Biosynthesis of cofactors, prosthetic groups, and carriers | ++ | - | - | - | - | - |
| Locus | Protein function | JCVI role category | Sensitivity to H_2_O_2_^a^ | Sensitivity to Antibiotic^b^ | Opsono- assay^c^ | Adhesion to CaCo2 | Virulence in  *G. mellonella*^d^ | Growth ability |
| EF3272 | zinc-binding transcriptional regulator, Cro/CI family | Regulatory functions | - | - | - | - | - | - |
| EF3279 | peptidase, U32 family | Protein fate | - | - | - | - | - | - |
| EF3280 | peptidase, U32 family, putative | Protein fate | - | - | - | - | Increased+ | - |
| EF3281 | conserved domain protein | Hypothetical proteins | - | - | - | - | - | - |
| EF3294 | membrane protein, putative | Cell envelope | - | - | - | - | - | - |

^a^ For resistance to oxidative stress + represents mutants slightly sensitive ++ highly sensitive and +++ extremely sensitive.

^b^ For antibiotic resistance tests AmoxRes means resistant to amoxicilline, CefSen sensitive to Cefoperazone, ChloramRes resistant to Chloramphenicol, CeftriSen++ highly sensitive to ceftriaxone, GenRes resistant to Gentamicin, pleiotrophic pleiotrophic effects of the mutation, FusidASen sensitive to fusidic acid, MetRes resistant to methicillin, MetSen sensitive to methicillin.

^c^ For opsonophagocytosis assay Ser-Ind, Co-Sens and High-Res means Serum Independent killing, Complement sensitivity and Higher Resistance, respectively.

^d^ For virulence in *G. mellonella* P values of <0.05 were considered statistically significant with the following classification: 0.005< P <0.05 (decreased or increased), 0.0005< P <0.005 (decreased+ or increased+) and P <0.0005 (decreased++ or increased++).
