# Supplementary material for: Large-Scale Screening of a Targeted Enterococcus faecalis Mutant Library Identifies Envelope Fitness Factors
Source: PLoS One. 2011 Dec 15;6(12):e29023. doi: 10.1371/journal.pone.0029023 (PMC3240637; doi:10.1371/journal.pone.0029023)
Supplement: Table S2 — Insertionally inactivated genes in mutants affected in oxidative stress response. (DOC) [file pone.0029023.s004.doc]

**Table S2.** List of the targeted genes of mutants affected in the oxydative stress response and JCVI role categories.

| JCVI role category | Locus | Protein function | Sensitivity to H_2_O_2_^a^ | Sensitization factor^b^ |
| --- | --- | --- | --- | --- |
| Biosynthesis of cofactors, prosthetic groups, and carriers | EF3255 | thiamin biosynthesis lipoprotein ApbE, putative | ++ | 0.72 |
| Cell envelope | EF0071 | lipoprotein, putative | +++ | 0.68 |
|  | EF0746 | penicillin-binding protein, putative | ++ | 0.71 |
|  | EF1027 | membrane protein, putative | + | 0.75 |
|  | EF1172 | teichoic acid biosynthesis protein B, putative | +++ | 0.55 |
|  | EF1173 | glycosyl transferase, WecB/TagA/CpsF family | +++ | 0.66 |
|  | EF1583 | N-acetylmuramoyl-L-alanine amidase, family 4 | ++ | 0.71 |
|  | EF1746 | UTP-glucose-1-phosphate uridylyltransferase | +++ | 0.64 |
|  | EF2170 | glycosyl transferase, group 2 family protein | +++ | 0.69 |
|  | EF2180 | glycosyl transferase, group 2 family protein | +++ | 0.66 |
|  | EF2195 | glycosyl transferase, group 2 family protein | + | 0.76 |
|  | EF2196 | glycosyl transferase, group 2 family protein | +++ | 0.64 |
|  | EF2198 | glycosyl transferase, group 4 family protein | +++ | 0.65 |
|  | EF2347 | cell wall surface anchor family protein | ++ | 0.74 |
| Cellular processes | EF0079 | gls24 protein | ++ | 0.74 |
|  | EF0080 | gls24 protein | ++ | 0.7 |
|  | EF0146 | surface exclusion protein, putative | ++ | 0.7 |
|  | EF0604 | gls24 protein | +++ | 0.69 |
| Energy metabolism | EF0590 | polysaccharide deacetylase family protein | ++ | 0.71 |
|  | EF1238 | glycosyl hydrolase, family 3 | +++ | 0.66 |
|  | EF1347 | glycosyl hydrolase, family 13 | ++ | 0.71 |
| Hypothetical proteins | EF1288 | conserved hypothetical protein | + | 0.77 |
|  | EF2268 | conserved hypothetical protein | + | 0.77 |
|  | EF2490 | conserved hypothetical protein | + | 0.76 |
|  | EF2671 | conserved hypothetical protein | ++ | 0.72 |
|  | EF2682 | conserved hypothetical protein | +++ | 0.63 |
| No Data | EF0392 | hypothetical protein | ++ | 0.71 |
| JCVI role category | Locus | Protein function | Sensitivity to H_2_O_2_^a^ | Sensitization factor^b^ |
|  | EF0573 | hypothetical protein | +++ | 0.63 |
|  | EF1420 | hypothetical protein | ++ | 0.74 |
|  | EF1798 | hypothetical protein | +++ | 0.68 |
|  | EF2020 | hypothetical protein | +++ | 0.58 |
| Protein fate | EF1681 | peptide methionine sulfoxide reductase | + | 0.76 |
| Protein synthesis | EF0201 | translation elongation factor Tu | +++ | 0.56 |
| Regulatory functions | EF0073 | transcriptional regulator, Cro/CI family | +++ | 0.68 |
|  | EF0107 | transcriptional regulator, Crp/Fnr family | +++ | 0.61 |
|  | EF0465 | transcriptional regulator | ++ | 0.7 |
|  | EF0814 | transcriptional regulator, GntR family | + | 0.78 |
|  | EF1212 | transcriptional regulator | + | 0.79 |
|  | EF1525 | transcriptional regulator, Fur family | +++ | 0.63 |
|  | EF1599 | TPR domain transcriptional regulator, Cro/CI family | +++ | 0.63 |
|  | EF1741 | catabolite control protein A | ++ | 0.73 |
| Transport and binding proteins | EF1408 | ABC transporter, ATP-binding protein | +++ | 0.69 |
|  | EF1493 | V-type ATPase, subunit I | ++ | 0.74 |
|  | EF1705 | phosphate-binding protein | +++ | 0.59 |
|  | EF1760 | cell division ABC transporter, permease protein FtsX, putative | + | 0.77 |
|  | EF1869 | permease, putative | ++ | 0.73 |
|  | EF1920 | C4-dicarboxylate anaerobic carrier | ++ | 0.73 |
|  | EF2221 | ABC transporter, substrate-binding protein | + | 0.79 |
|  | EF2442 | phosphate transporter family protein | ++ | 0.74 |
|  | EF2992 | major facilitator family transporter | +++ | 0.66 |
| Unknown function | EF3060 | secreted lipase, putative | + | 0.79 |
|  | EF3164 | PilB family protein | +++ | 0.68 |

^a^ + represents mutants slightly sensitive (0,79<SF<0,74), ++ highly sensitive (0,74<SF<0,7) and +++ extremely sensitive (SF<0,69).

^b^ Sensitization factors (SF) are shown. SF is defined as the ratio of the time difference (Δt) of the V583-derived strain (VE14089) and the mutant strain (SCO) to reach OD600 of 0.3 divided by the Δt ratio of the VE14089 and SCO to reach OD600 of 0.3 in the presence of a given concentration of H_2_O_2_ (5, 6 or 7 mM). The values are the means of at least three different experiments.
