# Supplementary material for: Large-Scale Screening of a Targeted Enterococcus faecalis Mutant Library Identifies Envelope Fitness Factors
Source: PLoS One. 2011 Dec 15;6(12):e29023. doi: 10.1371/journal.pone.0029023 (PMC3240637; doi:10.1371/journal.pone.0029023)
Supplement: Table S3 — Insertionally inactivated genes in mutants affected in antibiotic resistance. (DOC) [file pone.0029023.s005.doc]

**Table S3.** List of the targeted genes of mutants affected in antibiotic resistance and JCVI role categories.

| JCVI role category | Locus | Protein function | Sensitivity to Antibiotic^a^ | MIC  (µg/mL)^b^ |
| --- | --- | --- | --- | --- |
| Cell envelope | EF0389 | membrane protein, putative | AmoxRes | 256 |
|  | EF0516 | membrane protein, putative | AmoxRes | 128 |
|  | EF1746 | UTP-glucose-1-phosphate uridylyltransferase | FusidASen | 1 |
|  | EF2167 | glycosyl transferase, group 2 family protein | MetSen | 32 |
|  | EF2181 | glycosyl transferase, group 2 family protein | FusidASen | 1 |
|  | EF2197 | glycosyl transferase, group 2 family protein | GenRes | 32 |
|  | EF2890 | glycosyl transferase, group 1 family protein | GenRes | 16 |
|  | EF2891 | glycosyl transferase, group 1 family protein | MetRes | 128 |
| Cellular processes | EF0146 | surface exclusion protein, putative | AmoxRes | 256 |
|  | EF0818 | polysaccharide lyase, family 8 | CefSen | 32 |
| Energy metabolism | EF1211 | NADH peroxidase | CeftriSen++ | 1 |
|  | EF1347 | glycosyl hydrolase, family 13 | pleiotrophic effects |  |
|  | EF2889 | 2-hydroxy-3-oxopropionate reductase | CefSen | 32 |
| No Data | EF2796 | hypothetical protein | GenRes | 16 |
| Protein fate | EF2997 | peptidase, M20/M25/M40 family | ChloramRes | 16 |
| Regulatory functions | EF1525 | transcriptional regulator, Fur family | GenRes | 64 |
| Signal transduction | EF0541 | PTS system component, authentic frameshift | AmoxRes | 256 |
|  | EF0553 | PTS system, IID component | AmoxRes | 128 |
|  | EF1193 | DNA-binding response regulator VicR | ChloramRes | 32 |
| Transport and binding proteins | EF1759 | phosphate ABC transporter, phosphate-binding protein | GenSens | 2 |
|  | EF2992 | major facilitator family transporter | GenRes | 32 |

^a^ AmoxRes, means resistant to amoxicillin, FusidASen sensitive to fusidic acid, MetSen sensitive to methicillin, GenRes resistant to Gentamicin, MetRes resistant to methicillin, CefSen sensitive to Cefoperazone, CeftriSen++ highly sensitive to ceftriaxone, ChloramRes resistant to Chloramphenicol.

^b^ MIC values for VE14089 strain: Amoxicillin 4 µg/mL; Fusidic Acid 4 µg/mL; Methicillin 64 µg/mL; Gentamicin 8 µg/mL; Cefoperazone 64 µg/mL; Ceftriaxone 256 µg/mL; Chloramphenicol 4 µg/mL.
