# Supplementary material for: Large-Scale Screening of a Targeted Enterococcus faecalis Mutant Library Identifies Envelope Fitness Factors
Source: PLoS One. 2011 Dec 15;6(12):e29023. doi: 10.1371/journal.pone.0029023 (PMC3240637; doi:10.1371/journal.pone.0029023)
Supplement: Table S4 — Insertionally inactivated genes in mutants affected in resistance to opsonophagocytosis test. (DOC) [file pone.0029023.s006.doc]

**Table S4.** List of the targeted genes of mutants affected in resistance to opsonophagocytosis test and JCVI role categories.

| JCVI role category | Locus | Protein function | Classification of results | Complement induced phagocytosis^a^ | Phagocytosis with 1:5000 serum^c^ | Phagocytosis with 1:2500 serum^d^ | Complement only^e^ |
| --- | --- | --- | --- | --- | --- | --- | --- |
| Cell envelope | EF0176 | basic membrane protein family | Serum-independent killing | 91.3 | 97.9 | ns | ns |
|  | EF1027 | membrane protein, putative | Serum-independent killing | 80 | 86.4 | ns | ns |
|  | EF1172 | teichoic acid biosynthesis protein B, putative | Serum-independent killing | 89.4 | 97.6 | ns | ns |
|  | EF1173 | glycosyl transferase, WecB/TagA/CpsF family | Serum-independent killing | 88.8 | 95.4 | ns | ns |
|  | EF1746 | UTP-glucose-1-phosphate uridylyltransferase | Serum-independent killing | 85.1 | 89.8 | ns | ns |
|  | EF2167 | glycosyl transferase, group 2 family protein | Serum-independent killing | 88 | 93.1 | ns | ns |
|  | EF2170 | glycosyl transferase, group 2 family protein | Serum-independent killing | 89.9 | 92.5 | ns | ns |
|  | EF2196 | glycosyl transferase, group 2 family protein | Serum-independent killing | 82.8 | 90.4 | ns | ns |
|  | EF2198 | glycosyl transferase, group 4 family protein | Serum-independent killing | 94 | 92.2 | ns | ns |
|  | EF2890 | glycosyl transferase, group 1 family protein | Complement sensitivity | ns^b^ | ns | ns | 92.3 |
| Hypothetical proteins | EF2490 | conserved hypothetical protein | Higher resistance | ns | ns | 53.8 | ns |
| Regulatory functions | EF0600 | transcriptional regulator, TetR family | Higher resistance | ns | ns | 69.1 | ns |
|  | EF0601 | transcriptional regulator, TetR family | Higher resistance | ns | ns | 57.8 | ns |
| Transport and binding proteins | EF1705 | phosphate-binding protein | Serum-independent killing | 82.3 | 89.8 | ns | ns |

^a^Average killing percentage in Serum independent Complement-Induced phagocytosis (2.7% for VE14089)

^b^No significant difference between mutant and control strains

^c^Average killing percentage in phagocytosis with 1:5000 Serum (35.7% for VE14089)

^d^Average killing percentage in phagocytosis with 1:2500 Serum (86% for VE14089)

^e^Average killing percentage by complement only (3.1% for VE14089)
