# Supplementary material for: Large-Scale Screening of a Targeted Enterococcus faecalis Mutant Library Identifies Envelope Fitness Factors
Source: PLoS One. 2011 Dec 15;6(12):e29023. doi: 10.1371/journal.pone.0029023 (PMC3240637; doi:10.1371/journal.pone.0029023)
Supplement: Table S5 — Insertionally inactivated genes in mutants affected in the adhesion to Caco-2 cells. (DOC) [file pone.0029023.s007.doc]

**Table S5.** List of the targeted genes of mutants affected in the adhesion to CaCo2 cells and JCVI role categories.

| JCVI role category | Locus | Protein function | Adhesion to Caco2 | Adhesion ratio^a^ |
| --- | --- | --- | --- | --- |
| Cell envelope | EF0252 | N-acetylmuramoyl-L-alanine amidase, family 4 | decreased | 0.26 |
| Cellular processes | EF1076 | streptomycin 3-adenylyltransferase, putative | decreased | 0.32 |
| No Data | EF0876 | hypothetical protein with MGA helix turn helix domain | decreased | 0.21 |
| Signal transduction | EF0553 | PTS system, IID component | decreased | 0.34 |
| Cell envelope | EF0617 | membrane protein, putative | increased | 2.27 |
|  | EF0746 | penicillin-binding protein, putative | increased | 5.43 |
|  | EF0994 | UDP-N-acetylglucosamine--N-acetylmuramyl-(pentapeptide) pyrophosphoryl-undecaprenol N-acetylglucosamine transferase | increased | 2.12 |
|  | EF1027 | membrane protein, putative | increased | 3.08 |
|  | EF1172 | teichoic acid biosynthesis protein B, putative | increased | 2.8 |
|  | EF2167 | glycosyl transferase, group 2 family protein | increased | 2.05 |
|  | EF2196 | glycosyl transferase, group 2 family protein | increased | 6.48 |
|  | EF2525 | cell wall surface anchor family protein | increased | 2.66 |
| Cellular processes | EF0080 | gls24 protein | increased | 2.76 |
|  | EF0577 | adhesion lipoprotein | increased | 2.33 |
| Energy metabolism | EF1606 | glycosyl hydrolase, family 1 | increased | 1.95 |
|  | EF2556 | fumarate reductase flavoprotein subunit precursor, putative | increased | 1.77 |
| Hypothetical proteins | EF0086 | conserved domain protein | increased | 2.08 |
|  | EF0609 | conserved hypothetical protein | increased | 3.46 |
| No Data | EF2796 | hypothetical protein | increased | 3.29 |
| Protein synthesis | EF0201 | translation elongation factor Tu | increased | 2.58 |
| Regulatory functions | EF0579 | transcriptional regulator, putative | increased | 2.05 |
|  | EF0814 | transcriptional regulator, GntR family | increased | 1.88 |
|  | EF1525 | transcriptional regulator, Fur family | increased | 2.45 |
| Signal transduction | EF1516 | PTS system, IIABC components | increased | 2.75 |
| Transport and binding proteins | EF1705 | phosphate-binding protein | increased | 3.84 |
|  | EF1869 | permease, putative | increased | 1.92 |
|  | EF2772 | drug resistance transporter, putative, authentic frameshift | increased | 3.11 |
|  | EF2992 | major facilitator family transporter | increased | 1.76 |

^a^ The adhesion ratio was the percentage of mutant adherent bacteria divided by the percentage of VE14089 adherent bacteria from the same experiment. This calculation lowers the variation between independent experiments e.g. the number and confluence of eukaryotic cells. This ratio is equal to 1 if the mutant and VE14089 bacteria adhere similarly, >1 if the mutant adhere more, and <1 if the mutants adhere less than the VE14089. Values over 1.62 (increased) and under 0.37 (decreased) were considered significant.
