# Supplementary material for: Large-Scale Screening of a Targeted Enterococcus faecalis Mutant Library Identifies Envelope Fitness Factors
Source: PLoS One. 2011 Dec 15;6(12):e29023. doi: 10.1371/journal.pone.0029023 (PMC3240637; doi:10.1371/journal.pone.0029023)
Supplement: Table S6 — Insertionally inactivated genes in mutants affected in virulence towards Galleria mellonella . (DOC) [file pone.0029023.s008.doc]

**Table S6.** List of the targeted genes of mutants affected for virulence towards *Galleria mellonella* and JCVI role categories.

| JCVI role category | Locus | Protein function | Virulence in  *G. mellonella*^a^ |
| --- | --- | --- | --- |
| Cell envelope | EF0252 | N-acetylmuramoyl-L-alanine amidase, family 4 | Decreased++ |
|  | EF0362 | chitin binding protein, putative | Decreased++ |
|  | EF0887 | glycosyl transferase, group 2 family protein | Decreased++ |
|  | EF0994 | UDP-N-acetylglucosamine--N-acetylmuramyl-(pentapeptide) pyrophosphoryl-undecaprenol N-acetylglucosamine transferase | Decreased+ |
|  | EF1027 | membrane protein, putative | Decreased++ |
|  | EF1172 | teichoic acid biosynthesis protein B, putative | Decreased++ |
|  | EF1175 | glycerol-3-phosphate cytidylyltransferase | Decreased++ |
|  | EF1746 | UTP-glucose-1-phosphate uridylyltransferase | Decreased |
|  | EF2167 | glycosyl transferase, group 2 family protein | Decreased+ |
|  | EF2170 | glycosyl transferase, group 2 family protein | Decreased++ |
|  | EF2181 | glycosyl transferase, group 2 family protein | Decreased++ |
|  | EF2196 | glycosyl transferase, group 2 family protein | Decreased++ |
|  | EF2197 | glycosyl transferase, group 2 family protein | Decreased++ |
|  | EF2198 | glycosyl transferase, group 4 family protein | Decreased++ |
|  | EF2746 | dltD protein | Decreased++ |
|  | EF0818 | Polysaccharide lyase, Family 8 | Decreased |
| Cellular processes | EF2662 | choline binding protein | Decreased |
|  | EF1211 | NADH peroxidase | Decreased++ |
| Energy metabolism | EF1851 | glycosyl hydrolase, family 35 | Increased++ |
|  | EF3157 | glycosyl hydrolase, family 65 | Decreased++ |
|  | EF1663 | branched-chain phosphotransacylase | Decreased |
| Fatty acid and phospholipid metabolism | EF0086 | conserved domain protein | Decreased |
| Hypothetical proteins | EF0091 | conserved hypothetical protein | Increased++ |
|  | EF0906 | conserved hypothetical protein | Increased++ |
|  | EF2250 | conserved domain protein | Decreased++ |
| JCVI role category | Locus | Protein function | Virulence in  *G. mellonella*^a^ |
|  | EF0876 | hypothetical protein, MGA Helix-turn-helix domain | Decreased++ |
| No Data | EF1420 | hypothetical protein | Decreased++ |
|  | EF1798 | hypothetical protein | Decreased |
|  | EF2276 | hypothetical protein | Increased |
|  | EF2748 | peptidase, U32 family, putative | Decreased++ |
| Protein fate | EF2997 | peptidase, M20/M25/M40 family | Decreased+ |
|  | EF3280 | peptidase, U32 family, putative | increased+ |
|  | EF0073 | transcriptional regulator, Cro/CI family | Decreased+ |
| Regulatory functions | EF0403 | transcriptional regulator, MarR family | Decreased |
|  | EF0465 | transcriptional regulator | Decreased |
|  | EF0579 | transcriptional regulator, putative | Decreased++ |
|  | EF0600 | transcriptional regulator, TetR family | Decreased++ |
|  | EF0601 | transcriptional regulator, TetR family | Decreased++ |
|  | EF0814 | transcriptional regulator, GntR family | Decreased++ |
|  | EF1212 | transcriptional regulator | Decreased |
|  | EF1585 | transcriptional regulator, Fur family | Decreased++ |
|  | EF1741 | catabolite control protein A | Decreased++ |
|  | EF2417 | transcriptional regulator, Fur family | Decreased++ |
|  | EF3034 | transcriptional regulator, GntR family | Increased |
|  | EF0541 | PTS system component, authentic frameshift | Decreased+ |
| Signal transduction | EF1864 | DNA-binding response regulator | Decreased++ |
|  | EF0583 | ABC transporter, ATP-binding protein/permease protein | Increased+ |
| Transport and binding proteins | EF0785 | drug resistance transporter, EmrB/QacA family protein | Decreased+ |
|  | EF1493 | V-type ATPase, subunit I | Decreased |
|  | EF1592 | ABC transporter, ATP-binding/permease protein | Decreased+ |
|  | EF1705 | phosphate-binding protein | Decreased |
|  | EF1759 | phosphate ABC transporter, phosphate-binding protein | Decreased+ |
|  | EF1760 | cell division ABC transporter, permease protein FtsX, putative | Decreased |
| JCVI role category | Locus | Protein function | Virulence in  *G. mellonella*^a^ |
|  | EF1814 | drug resistance transporter, EmrB/QacA family protein | Decreased++ |
|  | EF2068 | multidrug resistance protein, putative | Decreased++ |
|  | EF2442 | phosphate transporter family protein | Decreased++ |
|  | EF2992 | major facilitator family transporter | Increased |

^a^ P values of <0.05 were considered statistically significant with the following classification: 0.005< P <0.05 (decreased or increased), 0.0005< P <0.005 (decreased+ or increased+) and P <0.0005 (decreased++ or increased++).
