# Supplementary material for: Large-Scale Screening of a Targeted Enterococcus faecalis Mutant Library Identifies Envelope Fitness Factors
Source: PLoS One. 2011 Dec 15;6(12):e29023. doi: 10.1371/journal.pone.0029023 (PMC3240637; doi:10.1371/journal.pone.0029023)
Supplement: Text S1 — Supplementary text describing additional materials and methods utilized in the microarray transcriptomic experiment and list of antibiotics used in this study. (DOC) [file pone.0029023.s009.doc]

**SI Materials and Methods**

RNA samples and isolation

The VE14089 strain was grown to an OD_600_ of 0.5 in BHI broth, and RNA was extracted from 3 mls of culture, using the Qiagen RNeasy kit as per the manufacturer’s instructions. Equal amounts of total RNA from each of 3 replicates were pooled for cDNA synthesis after RNA quality control tests.

RNA quality control

Immediately prior to cDNA synthesis, the purity and concentration of RNA samples were determined from OD_260/280_ readings using a dual beam UV spectrophotometer and RNA integrity was determined by capillary electrophoresis using the RNA 6000 Nano Lab-on-a-Chip kit and the Bioanalyzer 2100 (Agilent Technologies, Santa Clara, CA) as per the manufacturer’s instructions.

cDNA synthesis and labeling

First strand cDNA was synthesized from 10-15 µg of total RNA using the SuperScript II Double-Stranded cDNA Synthesis Kit (Invitrogen, Carlsbad CA) and Random Hexamer Primers (Promega, Madison WI) according to the manufacturers’ instructions. The RNA template was degraded by incubation in 0.25N NaOH at 65^o^C and cDNA was purified after neutralization using QIAquick columns (Qiagen, Valencia CA). cDNA (3-7 µg) was fragmented with DNase I (Promega) and then labeled with biotinylated dd-UTP using the Bioarray™ Terminal Labeling Kit (Enzo, Plymouth Meeting PA). Briefly, each sample of fragmented cDNA was incubated with 20 µl of 5X Reaction Buffer, 10 µl of 10X CoCl_2_, 1 µl of 100X Biotin-ddUTP, and 2 µl of 50X Terminal Deoxynucleotide Transferase for 1 hr at 37°C.

Oligonucleotide array hybridization and analysis

The labeled cDNA fragments were hybridized for hybridized for 16hr at 48^o^C to the Affymetrix GeneChip, SLARE1 [1]. Arrays were washed and stained with phycoerythrein-conjugated streptavidin (Invitrogen/Life Technologies, Carlsbad, CA, USA) on a Fluidics Station 450 (Affymetrix) according to the manufacturer’s recommended procedures. Fluorescence intensities were determined using a GCS 3000 7G high-resolution confocal laser scanner and GeneChip Operating System v1.4 (GCOS; Affymetrix). Quality control metrics for cDNA integrity, sample loading, and variations in staining were determined after standardization of each array by global scaling the average of the fluorescent intensities of all genes on an array to constant target intensity (TGT) of 250. Expression data were analyzed by statistical algorithms resident in GCOS [2,3,4]. Robust expression changes were identified as having GCOS-calculated fold changes > 2.0, GCOS-generated detection p-values < 0.05 for all arrays in at least one group, and t-test p-values < 0.05 or 0.01.

List of antibiotics used

Penicillin G, Ampicillin, Amoxicillin, Methicillin, Piperacillin, Imipenem, Ceftriaxone, Cefoperazone, Vancomycin, Teicoplanin, Bacitracin, Polimyxin B, Colistina, Gentamicin, Netilmycin, Kanamicin, Chloramphenicol, Tetracyclin, Tigecycline, Erythromycin, Spiramycin, Lincomycin, Nitrofurantoin, Ciprofloxacin, Clindamicin, Quinipristin/Dalfopristin, Linezolid, Sulphamethoxazole/Trimethoprim, Norfloxacin, Ofloxacin, Enrofloxacin, Rifampicin, Fusidic acid, Mupirocin.

References

1. McBride SM, Fischetti VA, Leblanc DJ, Moellering RC, Jr., Gilmore MS (2007) Genetic diversity among *Enterococcus faecalis*. PLoS ONE 2: e582.

2. Hubbell E, Liu WM, Mei R (2002) Robust estimators for expression analysis. Bioinformatics 18: 1585-1592.

3. Liu WM, Mei R, Di X, Ryder TB, Hubbell E, et al. (2002) Analysis of high density expression microarrays with signed-rank call algorithms. Bioinformatics 18: 1593-1599.

4. Lockhart DJ, Dong H, Byrne MC, Follettie MT, Gallo MV, et al. (1996) Expression monitoring by hybridization to high-density oligonucleotide arrays. Nat Biotechnol 14: 1675-1680.
